# Metamaterial‐Enhanced Solar‐Driven Processes for Energy Conversion and Water Treatment

**DOI:** 10.1002/advs.202508046

**Published:** 2025-07-21

**Authors:** Xuechen Jing, Zhehao Sun, Hang Yin, Kaili Liu, Yi‐Lun Chen, Shuwen Cheng, Zongyou Yin

**Affiliations:** ^1^ Research School of Chemistry The Australian National University Canberra Australian Capital Territory 2601 Australia

**Keywords:** AI‐assisted designs, CO_2_ reductions, metamaterials, organic pollutant degradations, photocatalysis, solar energy conversion, water treatment

## Abstract

To address global challenges in sustainable energy and water treatment, metamaterials have emerged as a transformative class of materials for solar‐driven photocatalysis. Through nanoscale engineering, these artificially structured materials enable precise manipulation of light–matter interactions and significantly enhance solar energy utilization beyond the limits of conventional photocatalysts. This review systematically summarizes recent progress in applying metamaterials to solar‐driven processes for energy conversion and water treatment, including photocatalytic CO_2_ reduction, water splitting for hydrogen generation, degradation of organic pollutants, and solar‐driven water evaporation for purification. Key enhancement mechanisms include localized surface plasmon resonance, photonic bandgap engineering, and improved charge separation via metamaterial and semiconductor heterojunctions, which collectively improve light absorption, charge separation and transfer, and surface reactivity. Practical challenges related to scalable fabrication, long‐term durability, and integration into real‐world systems are also examined. Finally, emerging directions, including AI‐assisted inverse design, structural chirality, and multifunctional hybrid architectures, are discussed as promising strategies to further advance metamaterial‐based photocatalysts in sustainable energy and environmental applications.

## Graphical abstract

This review explores how metamaterials revolutionize solar‐driven process for energy conversion and water treatment through enhanced light absorption, LSPR, charge separation, and bandgap engineering. By leveraging unique nanostructures and AI‐guided design, these engineered materials drive CO₂ reduction, water splitting, pollutant degradation, and water evaporation, offering promising solutions for sustainable energy and environmental applications.

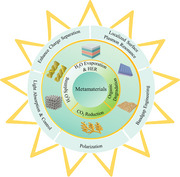

## 1 Introduction

Solar‐driven energy conversion and environmental remediation (such as photocatalytic fuel production and pollutant degradation) critically depend on efficient light harvesting and charge management in semiconductor materials.^[^ ^1^, ^2^ ^]^ Metamaterials are artificially engineered composites composed of sub‐wavelength structural units.^[^ ^3^ ^]^ They have recently gained significant attention for enhancing photocatalytic performance. Their unique electromagnetic properties, including intense resonant absorption and negative refractive index, originate primarily from carefully designed micro‐ and nanostructures rather than from intrinsic material chemistry.^[^ ^3^ ^]^ In other words, metamaterials are built from periodic or patterned “meta‐atoms” (e.g., metal or dielectric nanostructures) much smaller than the wavelength of light, enabling exotic optical behaviors not seen in natural materials.^[^ ^3^ ^]^ For example, appropriately designed metamaterials can bend and concentrate light in unconventional ways.^[^ ^4^, ^5^, ^6^, ^7^, ^8^, ^9^ ^]^ This allows them to achieve phenomena such as negative refraction or near‐perfect absorption of incident light, even in ultrathin layers. In contrast, conventional photocatalytic nanomaterials, such as oxide semiconductors or colloidal plasmonic nanoparticles, primarily depend on intrinsic material properties like bandgap or inherent plasmon resonance.^[^ ^10^, ^11^, ^12^ ^]^ They typically rely on random particulate assemblies to extend light‐path lengths or increase reactive surface areas. Metamaterials use structure‐driven optics to fundamentally alter this strategy. By engineering light–matter interactions through geometry rather than chemistry, they offer new pathways to manipulate light for photocatalysis beyond the capabilities of traditional materials.^[^ ^13^, ^14^ ^]^

One key light–matter interaction widely utilized in photocatalysis is localized surface plasmon resonance (LSPR).^[^ ^10^, ^15^, ^16^ ^]^ LSPR is a collective oscillation of conduction electrons within metal nanostructures. This phenomenon commonly occurs in noble metal nanoparticles and has long been used to enhance photocatalyst light absorption and create intense near fields.^[^ ^17^ ^]^ Many conventional plasmonic photocatalysts embed metal nanoparticles, such as Au and Ag, to exploit LSPR, thereby boosting optical absorption and generating energetic charge carriers for chemical reactions.^[^ ^18^, ^19^ ^]^ It should be emphasized, however, that LSPR is not unique to metamaterials; many colloidal metallic nanomaterials also exhibit this effect.^[^ ^17^ ^]^ What truly distinguishes metamaterials, including planar metasurfaces, is their ordered and coupled arrangement of plasmonic or dielectric building blocks, enabling collective resonances through intentional structural design. In a metasurface, coupling between neighboring nano‐resonators generates hybrid modes or collective oscillations.^[^ ^19^, ^20^ ^]^ These collective modes are more intense and tunable than the LSPR of an isolated nanoparticle. By carefully adjusting their geometry, metamaterials can control optical responses independently from the intrinsic properties of their materials.^[^ ^20^, ^21^ ^]^ Such control produces unique electromagnetic behaviors, including broadband absorption or sharp spectral selectivity, which single particles or random ensembles typically cannot achieve. Metamaterials can also support phenomena not found in natural materials, such as effective magnetic resonances and negative index refraction. These effects result directly from their engineered microstructures rather than their chemical composition. Unlike isolated nanoparticles, the ordered arrangement in metasurface designs produces stronger, more uniform electromagnetic field enhancements across the entire structure. By arranging plasmonic meta‐atoms into periodic arrays, metamaterials collectively exploit LSPR along with other resonant modes.^[^ ^19^, ^22^, ^23^, ^24^ ^]^ This structured approach maximizes the effective use of light in photocatalytic systems. Rather than merely absorbing more total light, metamaterials strategically concentrate electromagnetic energy at specific nanoscale sites where catalytic reactions occur. Such targeted energy localization significantly surpasses the capabilities of conventional plasmonic materials.

Creating these advanced optical structures involves fabrication methods different from traditional photocatalysts.^[^ ^15^ ^]^ Conventional nanomaterials, such as metal oxides or plasmonic nanoparticles, usually rely on bottom‐up methods like solvothermal growth or colloidal synthesis. These methods generally lack control over long‐range order or precise architecture. In contrast, metamaterials require accurately arranged nano‐units, typically fabricated using top‐down techniques.^[^ ^15^ ^]^ Electron‐beam lithography and focused ion beam milling, for example, are commonly employed to pattern metasurface resonators at the nanoscale. In photocatalytic applications, hybrid fabrication methods are often preferred. These methods combine precise top‐down patterning with bottom‐up synthesized functional nanocomponents.^[^ ^15^ ^]^ The goal is to incorporate metamaterials into photocatalytic systems, preserving both their optical functions (resonances, field enhancement) and surface chemical reactivity. This approach differs significantly from conventional catalyst designs, which mainly focus on tuning chemical composition or nanoparticle size and shape rather than constructing ordered nanostructures. Thus, metamaterial fabrication methods, from high‐resolution lithography to self‐assembly, represent a new strategy that integrates nanophotonic into catalyst design.

In the following sections, we systematically review how metamaterials are implemented in photocatalytic systems. The discussion emphasizes unique mechanisms and advantages compared to conventional methods. Several key enhancement strategies are detailed. These include improved broadband light absorption, enhanced reactions through near‐field LSPR effects, efficient charge separation in meta‐heterojunction structures, optimized reactivity from engineered surface interfaces, and polarization‐dependent photocatalysis using chiral metamaterials and chirality‐induced spin selectivity (CISS).^[^ ^25^, ^26^ ^]^ Each topic clearly distinguishes true metamaterial‐specific effects from those achievable with conventional nanomaterials. Examples from recent research highlight these distinctions. We then examine two major applications: photocatalytic CO₂ reduction and photocatalytic water treatment. These case studies demonstrate how metamaterial‐based designs significantly enhance performance and selectivity in practical chemical processes. Finally, we discuss practical considerations such as scalability, durability, and integration into devices. Emerging opportunities, including AI‐assisted inverse design and hybrid architectures, are also highlighted. This overview demonstrates how engineered metamaterials, with artificial electromagnetic properties and tailored interactions with light, drive photocatalysis toward greater efficiency and advanced functionalities.

## 2 Metamaterial‐Enabled Enhancement Mechanisms in Photocatalysis

### 2.1 Light Absorption and Control

Metamaterials offer a fundamentally different approach to light absorption than conventional photocatalyst designs. Rather than simply maximizing total absorption (which any black‐painted surface or dense colloidal film can do), metamaterial structures actively control how and where photons are absorbed, ensuring the energy is deposited in catalytically useful regions.^[^ ^27^, ^28^, ^29^, ^30^ ^]^ In fact, indiscriminate maximal absorption in a bulk material can be counterproductive when it causes extremely shallow photon penetration—a significant fraction of carriers might then be generated only at the very surface, where they can recombine or saturate without contributing to reactions deeper in the material. Metamaterials avoid this pitfall by decoupling optical absorption from the intrinsic properties and thickness of the material, using nanoscale architecture to manipulate light in ways conventional systems cannot. One key advantage is optical impedance matching through engineered refractive index profiles. Metamaterial absorbers (e.g., graded‐index metasurfaces) can gradually transition the effective index from that of air to that of the catalyst, virtually eliminating Fresnel reflections.^[^ ^30^, ^31^, ^32^, ^33^ ^]^ This means incoming light is channeled into the structure with minimal reflection over a broad spectrum and range of incidence angles, a feat not achievable with simple coatings or random nanoparticle dispersions. As a result, even a very thin metamaterial layer can capture as much light as a much thicker conventional film, but without the drawback of poor photon penetration depth.^[^ ^23^, ^30^ ^]^ The incoming photons are guided and distributed deeper into the active layer via the metamaterial's tailored gradient, ensuring that absorption occurs throughout the catalytically active volume rather than just at the immediate surface. Such impedance‐engineered metasurfaces thus combine high absorptance with effective photon delivery into the catalyst. Metamaterials also enable novel strategies for controlling and directing incident light, going beyond traditional media. They can guide and shape wavefronts, directing light into catalytic layers at optimal angles or with precise phase alignment.^[^ ^3^, ^5^, ^34^ ^]^ This creates constructive interference within the photocatalytic medium, enhancing overall absorption efficiency. Unlike conventional systems that rely on thick or highly absorbing layers, metamaterials manage incoming light more intelligently. They minimize reflection losses through impedance matching, trapping and recirculating photons to extend optical paths in thin films and concentrate energy directly at reactive nanoscale sites.^[^ ^35^ ^]^ In addition, metamaterials maintain high absorption over a broad range of incident angles. These sophisticated approaches allow metamaterials to improve photocarrier generation and utilization far beyond dispersed colloidal particles or conventional absorbing substrates. The result is not merely greater total light absorption, but significantly enhanced conversion efficiency, precisely delivering carriers where and when they are most effective for catalytic reactions.

### 2.2 Localized Surface Plasmon Resonance

**Figure 1:**
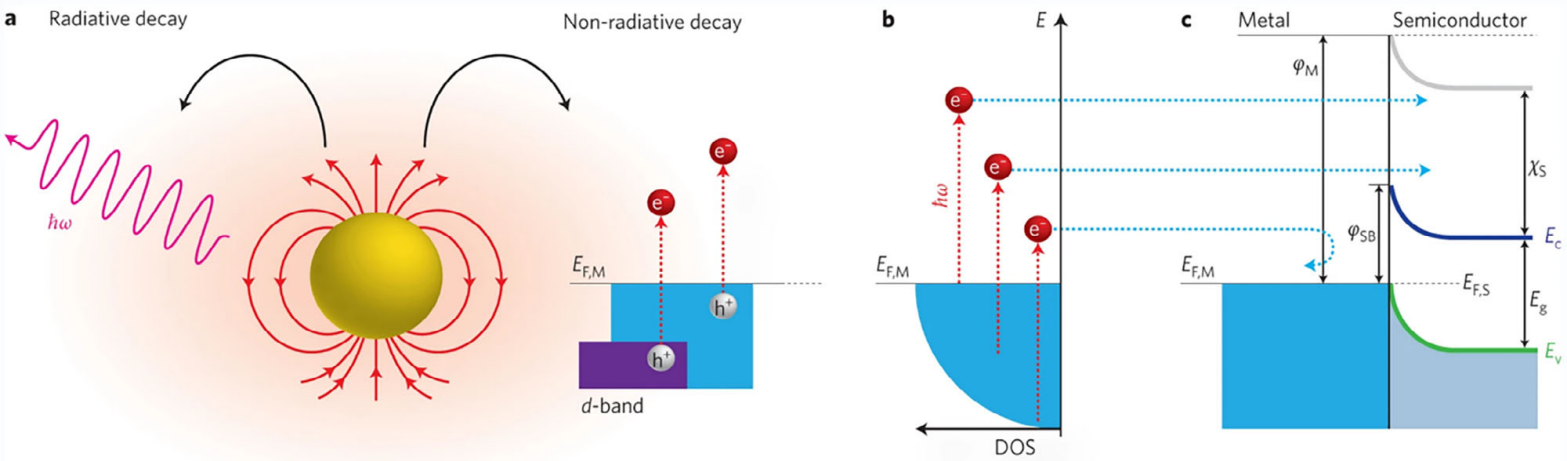
Schematic of plasmonic hot‐carrier generation and injection: a) radiative and nonradiative LSPR decay in a metal nanostructure, b) generation of energetic hot carriers above the Fermi level, and c) hot electron injection into a semiconductor across the Schottky barrier. Reproduced with permission.^[^ ^36^ ^]^ Copyright 2014, Springer Nature.

**Figure** **1** illustrates the fundamental mechanism of plasmonic hot‐carrier generation and injection.^[^ ^36^ ^]^ As depicted in Figure 1a, when LSPR is excited in a metal nanostructure, it can decay either radiatively (reemitting a photon) or nonradiatively via Landau damping, generating energetic “hot” electron–hole pairs. The nonradiative decay notably leads to a nonthermal distribution of electrons, with electrons elevated significantly above the Fermi level (*E_F_*), leaving hot holes behind, as shown in Figure 1b. These hot carriers, especially electrons with energies exceeding the Schottky barrier (*Φ_SB_*), can subsequently inject into the conduction band of an adjacent semiconductor, thereby initiating photocatalytic reactions (Figure 1c). LSPR is not unique to metamaterials.^[^ ^37^ ^]^ It occurs in suitably small metallic nanostructures such as Au, Ag, or Cu nanoparticles, appearing as a collective oscillation of conduction electrons coupled to incident light. The intense electromagnetic near‐fields generated around an excited plasmonic nanoparticle enhance light–matter interactions locally, and this basic near‐field enhancement occurs in conventional plasmonic catalysts as well.

The distinct advantage of metamaterials lies in how they spatially organize plasmonic units to collectively resonate, producing much stronger and more uniform electromagnetic effects than random nanoparticle dispersions. In carefully designed metamaterial architectures (e.g., periodic nanoparticle arrays or plasmonic superlattices), innumerable plasmons can coherently couple, yielding high‐density “hotspots” that cover a large area with relatively uniform field strength (variations within 10%). In contrast to isolated particles or haphazard clusters, which produce only isolated intense spots, a metamaterial can thus bathe a whole catalytic surface in enhanced near‐fields. This collective resonance approach enables not only stronger confinement of light but also precise control over the resonance frequency and spatial field distribution via the meta‐atom geometry and arrangement.^[^ ^38^ ^]^ Metamaterial designs excel at amplifying the electromagnetic near‐field associated with LSPR far beyond the levels in conventional systems. By incorporating nanoscale features like narrow gaps, periodic patterns, or multilayer structures, they can achieve exceptional field enhancement factors. For instance, plasmonic nanoparticle dimers and coupled arrays generate extraordinarily intense hotspots in their junctions.^[^ ^36^ ^]^ For example, a metal–insulator–metal nanocavity metasurface can achieve over 60% light absorption at resonance.^[^ ^39^ ^]^ This absorption is roughly five times higher than a bare nanoparticle film, with local field intensities increased by factors of 10^3^–10^5^ in nanoscale gaps.^[^ ^39^ ^]^ Such enormous field amplification corresponds to several orders of magnitude higher localized light intensity, effectively funneling electromagnetic energy into the vicinity of catalytic sites. The result is a dramatic increase in the generation of charge carriers and excitations in those regions, as the strongly confined optical energy is converted into electronic excitations (or directly into chemical activity) much more efficiently than in non‐metamaterial setups. Crucially, metamaterials can maintain this enhancement uniformly across the active area, avoiding the “hotspot lottery” of random nanoparticle aggregates and ensuring that a large fraction of the catalyst surface experiences intensified fields.

#### 2.2.1 Near‐Field Enhancement

The strong near field significantly increases the yield of hot carriers produced by plasmon decay. When LSPRs decay nonradiatively, the high local field intensity generates large numbers of energetic electrons and holes per unit volume in metal nanostructures.^[^ ^40^ ^]^ Metamaterials therefore serve as efficient hot‐carrier sources, achieving densities far higher than isolated nanoparticles of the same volume. These hot electrons and holes possess energies above the Fermi level, enabling them to participate in chemical reactions or transfer into neighboring materials. In metal–semiconductor metamaterial heterostructures, hot electrons are typically harvested by transferring across a Schottky barrier into the conduction band of the semiconductor (Figure 1c). This electron transfer directly initiates photocatalytic reactions. The energetic electrons provided by LSPR can overcome activation barriers and initiate chemical transformations that conventional photoexcited or thermalized electrons typically cannot achieve. By delivering these energetic carriers directly to catalytic sites, metamaterial‐enhanced LSPR effectively reduces activation energies and accelerates reaction kinetics beyond conventional photocatalytic methods.

#### 2.2.2 Photothermal Effect

In tandem with charge carriers, plasmonic photothermal effects also play a role in metamaterial‐enhanced catalysis. Nonradiative plasmon decay inevitably produces local heat in the metal nanostructure as the excited electrons relax, and in a metamaterial this heating can be both substantial and highly localized.^[^ ^18^ ^]^ The temperature in the nanoscale vicinity of an active site can rise, accelerating thermally activated reaction steps (much like conventional thermal catalysis, but confined to the nanoscale). Metamaterials take advantage of this effect by precisely confining heat at catalytic interfaces, avoiding unnecessary heating of the entire bulk system. Studies demonstrate that localized heating from plasmonic structures significantly accelerates reaction rates, achieving outcomes usually possible only at higher overall temperatures. Photothermal activation under these mild conditions can even alter reaction selectivity by favoring specific pathways enhanced by localized temperature increases at the nanoscale.

Metamaterial designs also enable tuning the balance between hot‐carrier generation and photothermal heating. In certain structures, photothermal effects are deliberately reduced, enhancing direct hot‐carrier transfer instead. A recent study involving an Au–Pt bimetallic plasmonic superlattice demonstrated this clearly.^[^ ^18^ ^]^ Researchers found that improved catalytic activity under illumination mainly resulted from enhanced electromagnetic fields activating the Pt catalytic sites.^[^ ^18^ ^]^ In contrast, contributions from plasmonic heating and direct electron transfer from Au to Pt were comparatively minor.^[^ ^18^ ^]^ Such precise control highlights another key advantage of metamaterials. They allow targeted structural engineering to optimize either electromagnetic field‐driven carrier generation or photothermal effects, depending on the reaction requirements.

Overall, compared with traditional plasmonic systems, metamaterials offer distinct advantages not only in the magnitude of enhancement but also in field distribution, tunability, and spectral matching.^[^ ^11^, ^41^ ^]^ Metamaterials achieve uniform electromagnetic field distributions, thereby avoiding the uneven hotspot distribution inherent in randomly dispersed nanoparticle systems. Moreover, by carefully tailoring meta‐atom size, shape, periodicity, and dielectric environment, metamaterials can precisely control the LSPR resonance wavelength, facilitating broad‐spectrum or even multi‐band responses. Such spectral flexibility, coupled with high‐quality‐factor (narrow‐bandwidth) collective modes (e.g., surface lattice resonances, SLRs), is difficult to attain with conventional nanoparticles. Consequently, metamaterials emerge as highly tunable, spectrally precise, and spatially uniform plasmonic enhancement platforms, greatly surpassing traditional randomized nanoparticle configurations.

### 2.3 Enhanced Charge Separation and Carrier Dynamics

**Figure 2:**
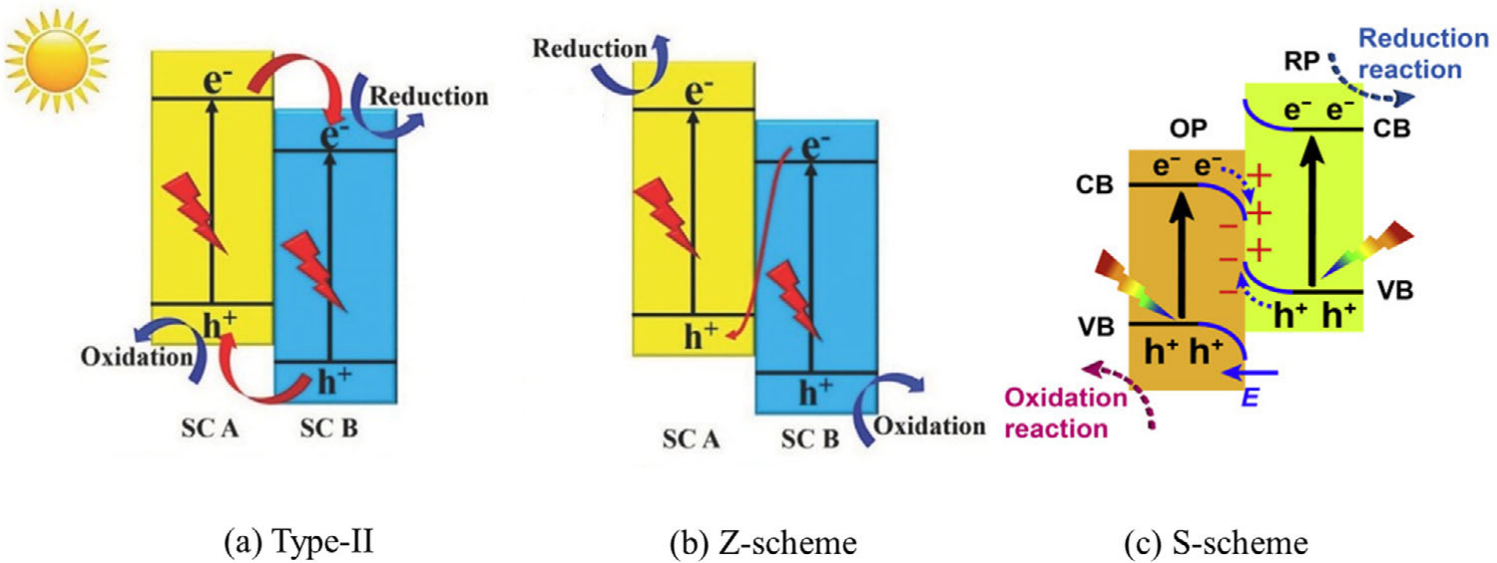
a) Type‐II heterojunction and b) Z‐scheme heterojunction Reproduced with permission. Copyright 2017, Wiley‐VCH.^[^ ^43^ ^]^ c) S‐scheme heterojunction. Reproduced with permission. Copyright 2020, Elsevier.^[^ ^44^ ^]^

One of the most critical challenges in photocatalysis is the rapid recombination of photogenerated electrons and holes, which severely undermines quantum efficiency. To overcome this, researchers engineer semiconductor heterojunctions with favorable band alignments and interfacial fields that drive electrons and holes into separate regions, thus spatially isolating reduction and oxidation sites. Metamaterial architecture allows these junctions to be tailored at the nanoscale, creating interface conditions that surpass what conventional materials can achieve.^[^ ^3^, ^42^ ^]^ Three major types of semiconductor heterojunctions have been explored for charge separation Type‐II, Z‐scheme, and the more recently developed S‐scheme, each offering a distinct charge transfer mechanism (**Figure** **2**).^[^ ^43^, ^44^, ^45^ ^]^ Below, we compare Type‐II and S‐scheme heterojunctions in technical detail and highlight how metamaterials uniquely leverage these designs to enhance photocatalysis.

Common heterojunction photocatalytic mechanisms include Type‐II, Z‐scheme and S‐scheme.^[^ ^45^ ^]^ Different mechanisms have different band structures and carrier transport paths. A conventional Type‐II heterojunction (staggered band alignment) relies on band offsets to separate charge carriers. In a Type‐II system, one semiconductor's conduction band (CB) is lower in energy than that of the other, so photogenerated electrons in the higher‐CB material will “flow” into the lower‐CB semiconductor, while holes migrate in the opposite direction into the higher‐valence‐band material (Figure 2a).^[^ ^44^ ^]^

This configuration physically separates electrons and holes (e⁻ accumulating in one semiconductor and h⁺ in the other), which reduces recombination and yields longer‐lived charges.^[^ ^45^ ^]^ However, this process reduces the electrochemical potential of each electron‐hole pair. Electrons transfer to a lower‐energy conduction band, while holes move to a higher‐energy valence band. In other words, Type‐II junctions sacrifice some driving force (redox power) in exchange for better carrier separation. This loss of carrier energy can compromise the system's ability to carry out strongly energetic redox reactions. By contrast, an S‐scheme (step‐scheme) heterojunction explicitly incorporates a built‐in electric field at the interface, achieving charge separation without forfeiting high carrier energies (Figure 2c).^[^ ^45^ ^]^ An S‐scheme is constructed by pairing a reduction‐type semiconductor (typically n‐type, with a relatively high CB position) with an oxidation‐type semiconductor (p‐type, with a deep valence band).^[^ ^45^ ^]^ When these two materials form a junction, electrons flow from one side to the other until their Fermi levels equilibrate, establishing a depletion region with a strong internal electric field at the interface.^[^ ^45^ ^]^ Under illumination, this field acts as a one‐way gate for charge carriers: it swiftly drives the less energetic electrons and holes toward each other to recombine in the interfacial region, while pushing the remaining high‐energy electrons and holes to the opposite outer sides of the heterojunction. In essence, the S‐scheme automatically disposes of the lowest‐energy electrons and holes (mimicking the electron–hole recombination step of a Z‐scheme) (figure 2b)) and preserves the most energetic electrons in the CB of the reduction‐side semiconductor and the most energetic holes in the VB of the oxidation‐side semiconductor.^[^ ^45^ ^]^ The outcome is a greatly suppressed recombination rate and maximized redox capacity, since each high‐energy carrier is now isolated on separate materials and can travel to a catalytic surface without encountering its opposite. S‐scheme heterojunctions are therefore attracting considerable attention because they combine the effective charge separation of Type‐II junctions with the strong redox potential of Z‐scheme systems, offering a clear performance advantage over traditional Type‐II designs.^[^ ^45^ ^]^

#### 2.3.1 Metamaterial‐Engineered versus Conventional Heterojunctions

Metamaterial architectures enhance heterojunction structures beyond conventional composite photocatalysts. Instead of merely placing two semiconductors together, metamaterials use precise nanoscale engineering. Examples include three‐dimensional periodic frameworks, nanopatterned interfaces, or ultrathin interlayers.^[^ ^42^, ^46^, ^47^ ^]^ These structures optimize conditions for light absorption and charge management at the junction. These engineered heterostructures can generate intense localized electric fields at the interface (due to enhanced band bending or embedded dipoles) and support strong LSPR if metallic “meta‐atoms” are included.^[^ ^24^ ^]^ They can also produce photothermal hotspots under illumination, raising the local temperature at the catalytic interface. All these metamaterial‐induced effects (nanoscale interface fields, LSPR, and photothermal heating) act in synergy to strengthen charge separation and carrier dynamics beyond what is achievable in a conventional heterojunction. For a traditional Type‐II heterojunction, incorporating metamaterial elements can partially offset its inherent limitations. For instance, integrating plasmonic metal nanostructures with a Type‐II semiconductor junction injects energetic “hot” electrons to boost the energy of photoexcited electrons that have cascaded into the lower band, and creates intense near‐field electromagnetic hotspots that assist in pulling apart electron–hole pairs.^[^ ^42^, ^48^ ^]^ These plasmonic effects help compensate for the lost redox potential by supplying additional energy to the charge carriers and by concentrating the electromagnetic field at the interface, leading to more efficient utilization of the separated charges. In essence, the metamaterial provides an external energy injection and a local driving force to a Type‐II system that would otherwise rely only on the modest band offset for charge separation.^[^ ^45^ ^]^

In S‐scheme heterojunctions, metamaterial enhancements become even more synergistic by reinforcing the strong internal electric field.^[^ ^42^, ^45^ ^]^ Structuring interfaces at the nanoscale, such as inserting a high‐permittivity dielectric or ferroelectric nanolayer at the p–n junction, further strengthens interfacial band bending. This effectively amplifies the built‐in electric field within the S‐scheme heterojunction. Meanwhile, plasmonic metamaterial components (e.g., metal nanoparticle arrays or nanostructured alloys) embedded at or near the interface concentrate the incident light right at the junction and generate localized fields, injecting additional hot carriers that are immediately swept into the desired semiconductor by the internal field. The photothermal effect in metamaterial heterostructures arises from strong light absorption in metal or defect‐engineered semiconductor components.^[^ ^49^ ^]^ This effect increases the local temperature of the catalyst, accelerating surface reaction kinetics and improving charge‐carrier extraction at reaction sites, this means photogenerated electrons and holes that have been separated by the internal electric field are rapidly consumed in redox reactions (due to the higher reaction rate at elevated temperature), further suppressing bulk recombination. Overall, by combining a built‐in internal field with metamaterial‐driven optical and thermal enhancements, S‐scheme metamaterial heterojunctions achieve a level of charge separation and utilization far beyond that of a conventional Type‐II junction. In summary, metamaterial‐enhanced heterojunction structures significantly improve charge separation and carrier dynamics in Type‐II and S‐scheme systems. S‐scheme metamaterial photocatalysts provide an ideal combination of long‐lived, well‐separated charge carriers while preserving sufficient energy for challenging redox reactions.

### 2.4 Bandgap Engineering

Beyond optimizing light collection, metamaterials also open new frontiers in bandgap engineering—tailoring the electronic structure of a photocatalyst to extend its active spectral range and improve charge utilization.^[^ ^42^ ^]^ Traditional bandgap engineering in photocatalysis relies on methods like introducing dopants or defects to create mid‐gap states, forming semiconductor alloys or heterojunctions to tune band positions, or utilizing quantum confinement in nanostructures to adjust energy levels.^[^ ^49^ ^]^ While these approaches have seen success, they are often limited by material constraints: random dopant distributions can introduce recombination centers, quantum dot sizes are hard to perfectly control and integrate, and simple heterojunctions only provide a couple of discrete band alignments. Metamaterial platforms overcome many of these limitations by enabling precise, nanoscale control of composition and electromagnetic environment, unlocking bandgap‐modulation mechanisms that conventional materials cannot easily achieve. One powerful capability of metamaterial architectures is the creation of structured confinement and periodic potentials that modify a material's electronic band structure.^[^ ^5^, ^15^, ^50^ ^]^ By embedding a semiconductor into a metamaterial matrix or patterning it at sub‐wavelength scales, one can impose an artificial periodicity or confinement that leads to quantization and miniband formation. For instance, a metamaterial composed of alternating^[^ ^51^ ^]^ ultrathin layers or an array of semiconductor nanocrystals can act analogously to a superlattice, where charge carriers experience a new periodic potential.^[^ ^30^, ^52^ ^]^ This can result in quantum confinement effects finely tuned by geometry, the bandgap can be incrementally adjusted by the sizes of the features (e.g., nanopillar diameter or well thickness) with a level of uniformity and spatial order unattainable in a random quantum dot colloid.^[^ ^53^ ^]^ Metamaterial fabrication allows all the quantum‐confined units to be nearly identical and coherently coupled, so instead of a broad distribution of bandgap energies as in a typical nanoparticle ensemble, one gets a well‐defined, sharply tuned effective bandgap for the whole system. Moreover, coupling between these confined units through the metamaterial's periodic structure can create allowed miniband transitions within the bandgap that broaden the absorption spectrum. In essence, the metamaterial can turn isolated localized states into an extended intermediate band, permitting sub‐bandgap photons to be absorbed via two‐step electronic transitions—something extremely difficult to control in a conventional bulk‐doped or quantum‐dot system. Metamaterials also enable precise band alignment and multi‐component integration in ways that traditional heterostructures cannot. Rather than a simple planar junction between two semiconductors, a metamaterial can intertwine different semiconductors or a semiconductor and a plasmonic metal in a finely featured pattern (e.g., a 3D gyroid network or a core‐shell metasurface array). This level of architecture allows engineered band alignment at the nanoscale across a vast interface area, ensuring that photogenerated electrons and holes are immediately funneled into complementary materials or sites that facilitate their separation and use. Importantly, such metamaterial composites can achieve energy‐level configurations that yield new absorption pathways: for instance, deliberately introduced defect bands or dopant states can be coupled via the metamaterial's structure to a conduction band of another component, effectively creating a built‐in stepwise ladder for electrons. A photon with energy too low to directly cross the full semiconductor bandgap can still be absorbed. It first promotes an electron into a dopant‐induced intermediate state, and then further into the conduction band. This process effectively extends absorption to lower energies without lowering the semiconductor's band edges required for catalytic reactions.^[^ ^50^, ^51^ ^]^ In a conventional catalyst, these intermediate states often act as traps, but in a metamaterial, they can be arranged and coupled in a regular way, turning them into productive pathways rather than dead ends. Metamaterials can also create smooth variations in composition, producing graded bandgap structures throughout the catalyst. This design broadens absorption and generates internal electric fields, achieving a level of precision difficult to obtain using conventional bulk synthesis methods. Perhaps the most striking opportunities emerge from the hybridization of electronic states with photonic modes in metamaterials. Because metamaterials can strongly confine and enhance electromagnetic fields, they allow the regime of strong light matter coupling, where a semiconductor's excitonic transition (or a molecule's electronic transition) can mix with a resonant photonic mode to form new hybrid states (polariton modes). These hybrid light matter states have energies and selection rules distinct from the original exciton or band transition. In practical terms, a metamaterial supporting such mode hybridization can split a single absorption band into two lighter and lower energy coupled bands, effectively expanding the range of photon energies that can excite the system. For example, coupling electronic transitions of a wide bandgap semiconductor with a plasmonic or Fabry–Pérot resonator mode can generate two polariton absorption peaks.^[^ ^51^, ^54^, ^55^, ^56^ ^]^ One peak can even appear below the semiconductor's original bandgap energy. Photons that would normally be too weak to excite an electron across the bandgap can instead be absorbed into the lower hybrid mode, still resulting in an excited electron that can participate in chemistry (via mechanisms like hot‐electron injection or sensitized excitation). This extended absorption occurs within the material's own electronic framework. The new absorption channel arises directly from a polaritonic state, without relying on external sensitizers or multi‐photon processes. Metamaterials thus provide a platform for quantum photonic coupling that shifts or effectively narrows the bandgap from the perspective of usable sunlight, all while maintaining the catalyst's bulk chemical identity. This kind of tailored strong coupling or dispersion engineering (for instance, using hyperbolic metamaterials to provide an enormous range of electromagnetic wavevectors) has no real analogue in a traditional photocatalyst.^[^ ^54^, ^55^ ^]^ Conventional materials rarely support these coupling regimes or the necessary nanostructures. However, metamaterials are specifically designed to exploit them. In summary, metamaterial‐based bandgap engineering equips photocatalysts with electronic and optical functionalities well beyond the reach of conventional methods. By introducing finely tuned dopant arrays, quantum‐confined architectures, and hybrid light matter states, metamaterials allow the photocatalyst to harvest lower‐energy photons and generate charge carriers that would otherwise be inaccessible. These platforms precisely control energy levels and electronic transitions. For example, they transform defect states into beneficial intermediate bands or use unusual dispersion modes, such as hyperbolic modes, to facilitate indirect transitions. This approach broadens the effective absorption spectrum without sacrificing charge separation or catalytic redox potentials. Crucially, these changes are integral to the material: the extended light response comes from engineered band structures and couplings inside the catalyst, not from adding external dyes or excessively narrowing the bandgap.

### 2.5 Polarization‐Dependent Photocatalysis

Embedding structural chirality into metamaterial photocatalysts induces asymmetric catalytic behavior not achievable with achiral systems. Chiral structures, such as plasmonic films or nanoarrays with helical patterns, selectively interact with circularly polarized light (CPL).^[^ ^25^, ^57^, ^58^ ^]^ This selective interaction results in polarization‐dependent improvements in reaction rates.

Experiments have shown that matching the incident light's chirality with that of a chiral metamaterial can dramatically boost photocatalytic performance, whereas the opposite handedness yields diminished activity.^[^ ^58^, ^59^, ^60^ ^]^ In one case, a chiral plasmonic film degraded dye molecules roughly twice as fast under CPL of the “matching” helicity compared to CPL of the opposite sense.^[^ ^58^ ^]^ This behavior arises from the chiral structure's ability to concentrate electromagnetic fields and excited charges (e.g., hot electrons) more efficiently under the resonant polarization, creating a dissymmetric charge generation and local “superchiral” optical fields that favor one spin or orientation.^[^ ^58^ ^]^ Such effects effectively make the catalysis enantioselective with respect to light polarization: the two enantiomorphic forms of the catalyst (or illumination) drive reactions at different rates.^[^ ^58^ ^]^ Chiral metamaterial catalysts can even induce enantiospecific bias in chemical reactions. They preferentially accelerate the formation of one chiral product or favor the adsorption of one reactant enantiomer. This degree of selectivity is not achievable with conventional symmetric photocatalysts.

Beyond optical selection rules, CISS provides a further mechanism to amplify photocatalytic performance in these metamaterials.^[^ ^61^ ^]^ The CISS effect entails that electrons traversing a chiral medium become spin‐polarized, with one spin orientation transmitted preferentially due to the medium's helical electric field and spin–orbit coupling.^[^ ^59^, ^61^ ^]^ Incorporating such spin‐selective behavior into a photocatalytic process can substantially improve charge dynamics and reaction kinetics. Spin‐polarized charge carriers are less prone to recombination because identical‐spin electrons and holes cannot readily pair (a Pauli exclusion effect), forcing radiative or back‐reactions to slow down.^[^ ^25^ ^]^ This prolongs the lifetime of photogenerated carriers and increases their diffusion to catalytic sites. Moreover, many multi‐electron reactions benefit from spin alignment. For instance, in the oxygen evolution reaction (OER), intermediate oxygen radicals must share the same spin to form triplet O_2_ (the stable ground state); if spins are random, the pathway to O_2_ is kinetically hindered.^[^ ^25^ ^]^ A chiral catalyst that outputs spin‐polarized electrons can satisfy such requirements and lower activation barriers. Leveraging CISS has delivered clear performance gains. For instance, a hematite photoanode coated with chiral molecules exhibited higher OER currents and reduced peroxide side‐products compared to achiral control.^[^ ^25^ ^]^ In chiral metamaterials, this spin‐selective charge transport works in tandem with the intense local fields and optical chirality: the metamaterial not only harvests and concentrates light in an asymmetrical fashion but also channels the resulting charges in a spin‐aligned, more reactive form.^[^ ^25^, ^57^, ^60^, ^61^, ^62^ ^]^ Such synergy effectively serves as an internal spin filter or bias that directs charge carriers into favorable reaction pathways while impeding loss channels.^[^ ^25^ ^]^ In summary, structural chirality in metamaterials provides two key advantages. First, it enables polarization‐tuned interactions between light and matter for selective excitation. Second, through the CISS effect, it introduces spin‐polarized charge dynamics. Together, these effects significantly enhance photocatalytic activity, selectivity, and efficiency beyond what non‐chiral systems can achieve.

## 3 Metamaterials for CO_2_ Reduction

The efficient utilization of solar energy to drive CO_2_ reduction into value‐added fuels is a highly significant strategy for both environmental sustainability and renewable energy conversion.^[^ ^63^, ^64^ ^]^ The CO_2_ reduction reaction (CO_2_RR) involves multiple possible products depending on the number of electron‐proton transfer steps. Common C_1_ products include carbon monoxide (CO), formic acid (HCOOH), methanol (CH_3_OH), and methane (CH_4_), corresponding to two‐electron, six‐electron, and eight‐electron pathways, respectively.^[^ ^65^, ^66^, ^67^, ^68^ ^]^ The actual reaction pathway and product selectivity are strongly influenced by the surface structure, charge transfer pathways, and local reaction microenvironments of the catalyst.^[^ ^69^, ^70^ ^]^ Recent advances in metamaterial‐based photocatalysts offer unique opportunities to precisely modulate light harvesting, local electric fields, and charge carrier dynamics at the nanoscale, thus enabling the regulation of intermediate adsorption configurations and reaction barriers to steer product selectivity. Photothermal‐driven metamaterial absorbers have emerged as a powerful platform to lower reaction barriers and bias CO₂RR toward desired products.

**Figure 3:**
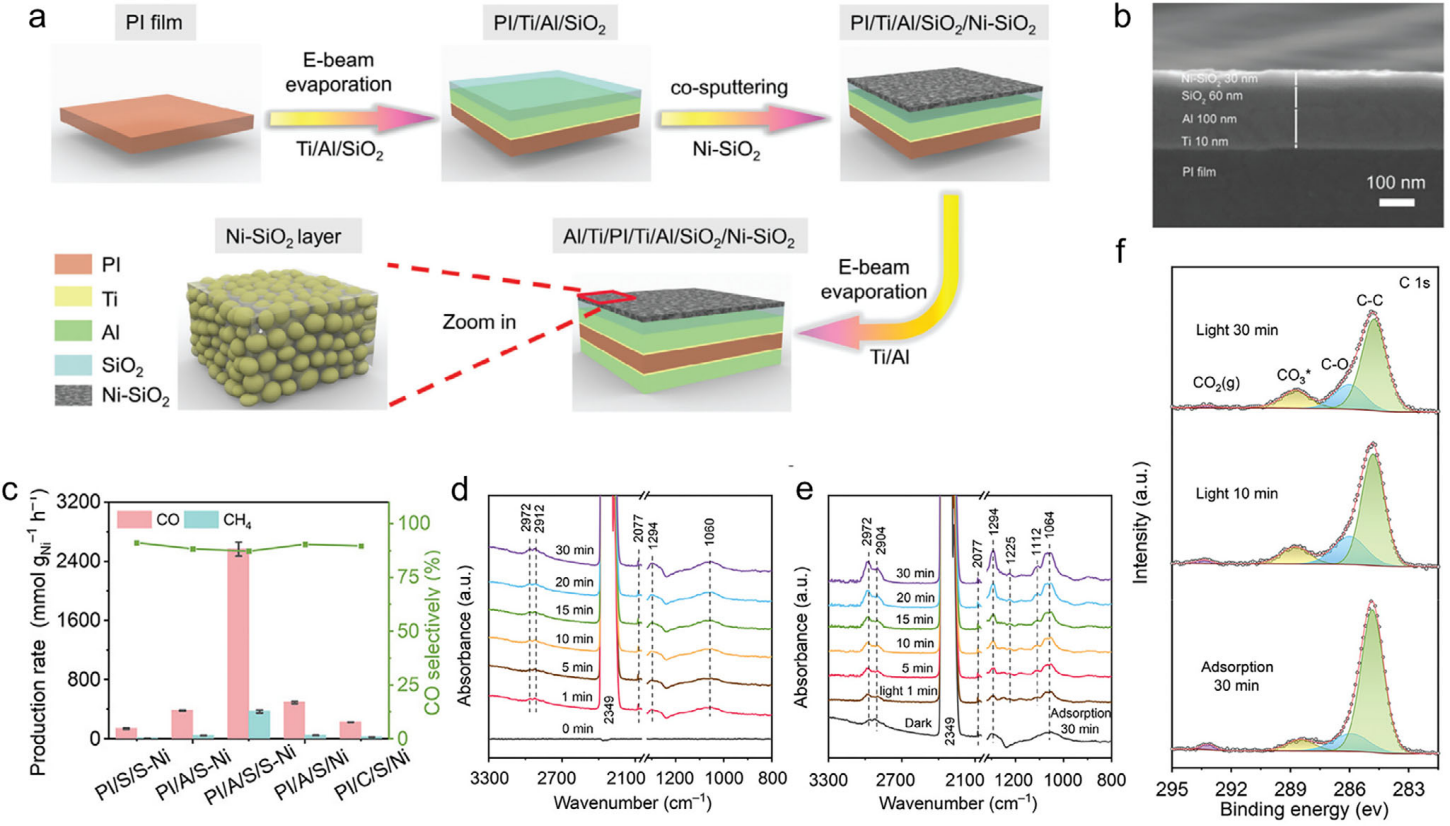
a) Schematic illustration of the preparation process for the PI/Ti/Al/SiO_2_/Ni‐SiO_2_ multilayer structure. Reproduced with permission.^[^ ^71^ ^]^ Copyright 2024, Wiley‐VCH. b) Side‐view scanning electron microscopy (SEM) image of the cross‐sectional structure of the PI/Ti/Al/SiO_2_/Ni‐SiO_2_ film. Reproduced with permission.^[^ ^71^ ^]^ Copyright 2024, Wiley‐VCH. c) Catalytic performance of PI/A/S/S‐Ni and control samples under 0.8 W cm^‐^^2^ irradiation. Reproduced with permission.^[^ ^71^ ^]^ Copyright 2024, Wiley‐VCH. d) In situ DRIFTS spectra of PI/A/S/S‐Ni under dark conditions, revealing adsorbed intermediates during CO₂ activation. Reproduced with permission.^[^ ^71^ ^]^ Copyright 2024, Wiley‐VCH. e) In situ DRIFTS spectra under light irradiation, illustrating the formation and conversion of reaction intermediates during photothermal CO₂ hydrogenation. Reproduced with permission.^[^ ^71^ ^]^ Copyright 2024, Wiley‐VCH. f) In situ C 1s near‐ambient‐pressure X‐ray photoelectron spectroscopy (NAP‐XPS) spectra of PI/A/S/S‐Ni during CO₂ hydrogenation. Reproduced with permission.^[^ ^71^ ^]^ Copyright 2024, Wiley‐VCH.

Photothermal catalysis holds great potential for CO_2_ hydrogenation.^[^ ^22^, ^42^, ^71^, ^72^, ^73^, ^74^, ^75^ ^]^ However, it typically requires high‐intensity light to achieve the temperatures necessary to break the stable CO_2_ bonds, which raises the complexity and energy consumption of the system. Reducing the light intensity requirement for practical applications is a key challenge in this field. To address this, Liu et al. designed a Ni‐based selective metamaterial absorber aimed at improving the efficiency of photothermal CO_2_ hydrogenation.^[^ ^71^ ^]^ The metamaterial consists of a polyimide (PI) substrate, a reflective Al layer, a dielectric SiO_2_ spacer layer, and embedded Ni nanoparticles (NPs) in SiO_2_. **Figure** **3**a provides a schematic illustration of the preparation process for PI/A/S/S‐Ni, accompanied by a side‐view scanning electron microscopy (SEM) AI image that offers detailed insights into the surface morphology and structure of the sample (Figure 3b). By optimizing solar absorption and minimizing thermal radiation loss, this design significantly increased local photothermal temperature under 0.8 W cm^−2^ light conditions. The plasmonic resonance effect of the Ni nanoparticles enhanced the local electric field, promoting the adsorption and activation of reactants. Experimental results indicate a CO_2_ conversion rate of 516.9 mmol g^−1^ h^−1^, with a CO selectivity of nearly 90%, maintaining stability over 10 h (Figure 3c). This performance surpassed conventional systems that require higher light intensity. Moreover, the morphology of SiO_2_‐coated Ni NPs improved Ni dispersion, enhanced contact with reactants, and prevented nanoparticle agglomeration at high temperatures, thus improving both activity and stability. In terms of thermal management, the metamaterial absorber reduced the emissivity at 300 °C to 0.08, significantly reducing thermal radiation losses. This efficient thermal management increased local temperatures without added energy consumption, demonstrating the important role metamaterials play in achieving high solar‐to‐chemical energy conversion efficiency. Mechanistic studies indicated that the enhanced catalytic performance was due not only to the increased temperature but also to nonthermal effects. The plasmon‐enhanced local electric field promoted the adsorption and activation of CO_2_ molecules. In situ near‐ambient‐pressure X‐ray photoelectron spectroscopy (NAP‐XPS) (Figure 3f) and diffuse reflectance infrared Fourier transform spectroscopy (DRIFTS) (Figure 3d,e) analyses revealed that under light irradiation, the intensity of surface carbonate species (XPS peak at 288.8 eV and DRIFTS bands at 1060 and 1294 cm⁻¹) significantly increased, indicating an enhanced chemisorption of CO₂ on the catalyst surface. This enhancement is attributed to the synergistic effect of localized photothermal heating and electric field‐induced activation, validating the interplay between light and thermal management in the catalytic process.

**Figure 4:**
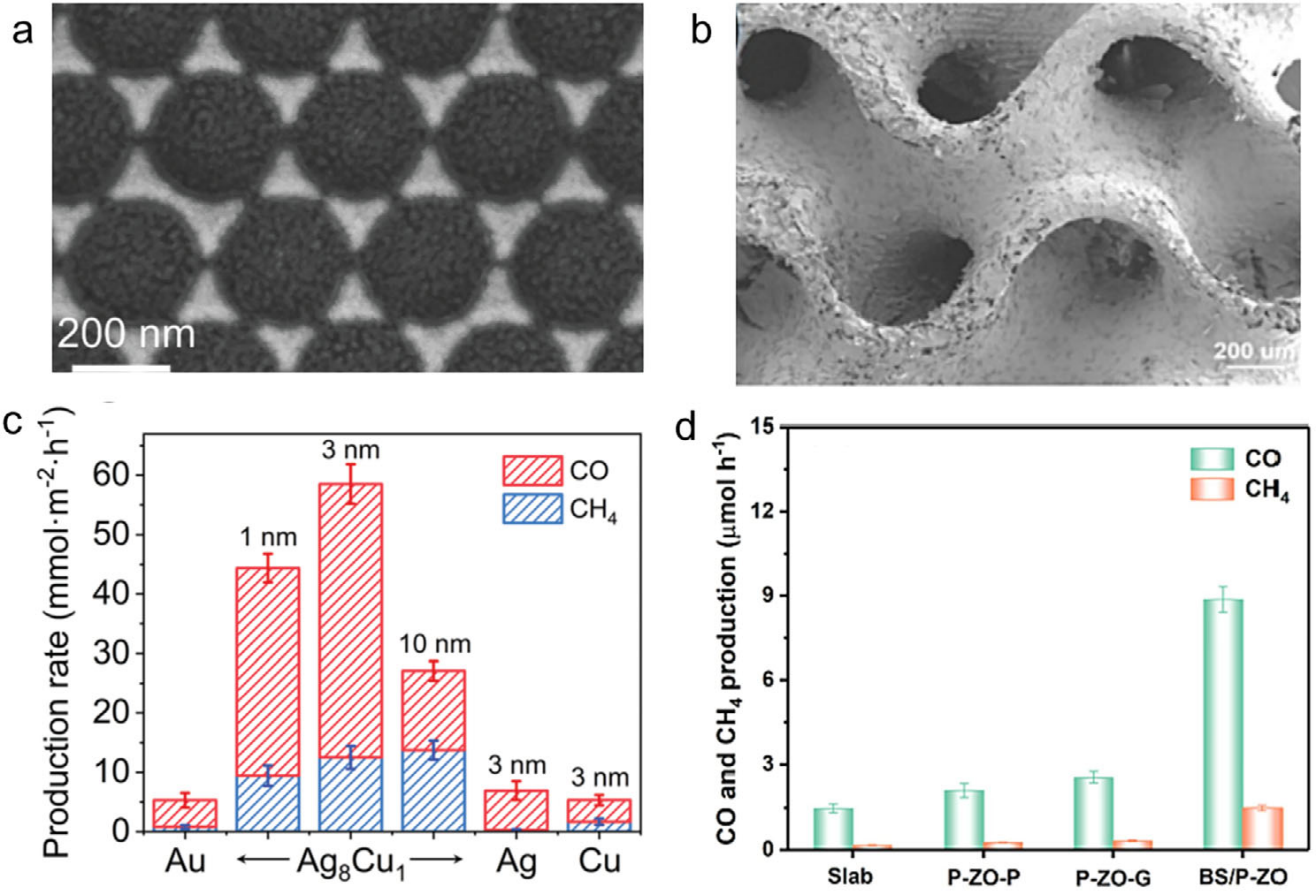
a) Top‐view SEM image of ASA‐c‐Ag_8_Cu_1_ triangle nanoarray. Reproduced with permission.^[^ ^76^ ^]^ Copyright 2022, Wiley‐VCH. b) SEM image of the B_2_S_3_/P‐ZnO gyroid metamaterials. Reproduced with permission.^[^ ^42^ ^]^ Copyright 2023, Elsevier. c) Production rates of CO and CH_4_ in catalytic CO_2_ hydrogenation using the ASA‐c‐Ag_8_Cu_1_ catalyst, compared to control samples under full‐spectrum light irradiation. Reproduced with permission.^[^ ^76^ ^]^ Copyright 2024, Wiley‐VCH. d) Production rate of CO and CH_4_ of B_2_S_3_/P‐ZnO. Reproduced with permission.^[^ ^42^ ^]^ Copyright 2023, Elsevier.

To further improve CO_2_ hydrogenation efficiency, Shao et al. developed a stacked plasmonic metamaterial that uses strong localized electric fields to drive efficient CO_2_ hydrogenation under broadband light.^[^ ^76^ ^]^ The metamaterial consists of a stacked Au‐SiO_2_‐Au‐c‐Ag_8_Cu_1_ trilayer structure as **Figure** **4**a shows (top view), enhancing light absorption across the broad spectral range of 370–1040 nm. This structure not only facilitates the generation of high surface temperatures required for photothermal catalysis but also induces strong localized electric fields at the surface, promoting hot electron transfer and lowering the activation barrier for CO_2_ hydrogenation. The combination of photothermal and plasmonic effects enabled efficient utilization of solar energy and heat generation. The plasmonic effect enhanced the activation of CO_2_ molecules, facilitating electron transfer at catalytic sites. Experimental results (Figure 4c) show CO and CH_4_ production rates of 1106 and 301 mmol m^−2^, respectively, over 24 h, with corresponding turnover frequencies (TOFs) of 1253 h^−1^ (CO) and 340 h^−1^ (CH_4_). The localized electric field played a key role in promoting the adsorption of intermediates such as *COOH and *CO on Cu sites within the Ag_8_Cu_1_ alloy. The field enhanced the selectivity of the reaction, with the (220) plane favoring CH_4_ formation and the (111) plane favoring CO formation. This activity difference was attributed to the localized electric field altering adsorption energies and thus influencing product selectivity. This metamaterial demonstrated excellent thermal stability, showing little degradation after 72 h of continuous operation. In addition, it was scalable through colloidal lithography, indicating its potential for large‐scale CO_2_ reduction systems.

Pan et al. developed a novel 3D Bi_2_S_3_/P‐ZnO gyroid metamaterial (Figure 4b),^[^ ^42^ ^]^ constructing an S‐scheme heterojunction to enhance CO_2_ reduction under simulated sunlight. This metamaterial was fabricated through 3D printing, followed by hydrothermal growth of Bi_2_S_3_ nanostructures on a P‐doped ZnO framework. The gyroid structure offers high surface area and interconnected pores, providing abundant active sites for CO_2_ reduction. The bionic villi‐like structure of Bi_2_S_3_ enhanced light scattering and absorption, generating multiple reflections within the structure and maximizing light utilization. P‐ZnO, with its oxygen vacancies, exhibited strong photothermal effects and a reduced bandgap, extending light absorption into the visible region. The combination of Bi_2_S_3_ and P‐ZnO amplified the photothermal effect, increasing surface temperature under illumination and promoting the separation of photogenerated carriers, thus enhancing CO_2_ adsorption and reduction. The S‐scheme heterojunction formed between Bi_2_S_3_ and P‐ZnO played a critical role in charge transfer. Unlike traditional type‐II heterojunctions, the S‐scheme heterojunction effectively separated and transferred photogenerated carriers while maintaining a high reduction potential for electrons and a high oxidation potential for holes. This mechanism led to increased yields of CO and CH_4_ during CO_2_ reduction. Experimental results from Figure 4d shows that the Bi_2_S_3_/P‐ZnO gyroid metamaterial exhibited significantly higher photocatalytic CO_2_ reduction performance than its individual components, achieving CO and CH_4_ production rates of 8.87 and 1.49 µmol h^−1^, respectively, under simulated sunlight. These values represent a 3.45‐fold and 4.65‐fold increase in CO and CH_4_ production compared to P‐ZnO alone. Enhanced light absorption, coupled with photothermal effects, hierarchical structure, and S‐scheme charge transfer, contributed to the superior catalytic performance. Mechanistic studies using in‐situ Fourier transform infrared spectroscopy (FTIR) and electrochemical analyses revealed that the reduction pathway involved intermediates such as CO_3_2^−^ and *COOH. The photothermal effect accelerated the conversion of these intermediates into CO and CH_4_. Efficient charge carrier separation reduced recombination rates, improving product selectivity and reaction rates.

In summary, metamaterial‐based catalysts offer unique avenues to tune CO_2_RR selectivity by leveraging their physical properties. By engineering the nanostructure and composition, metamaterials can modify the catalyst's band structure and electronic states, thereby influencing the binding energies of key intermediates. Plasmonic resonances in metamaterials create intense localized electric fields and can generate energetic “hot” electrons and holes under illumination, which preferentially drive certain reaction steps or lower the activation barriers for specific products. Moreover, light absorption by metamaterials leads to photothermal heating, elevating the local reaction temperature and potentially shifting the reaction equilibrium toward desired products. These combined effects enable metamaterial catalysts to steer the CO_2_ reduction reaction toward selectivity for target products.

## 4 Metamaterials for Water Splitting

The demand for efficient and sustainable hydrogen production has propelled research into photocatalytic water splitting. Recent advancements on metamaterials have focused on leveraging plasmonic effects, strong coupling phenomena, and tailored light absorption to enhance the photocatalytic activity of materials used in water splitting. This section reviews notable developments in metamaterial designs that have significantly improved hydrogen evolution reactions (HER) through photocatalysis.

**Figure 5:**
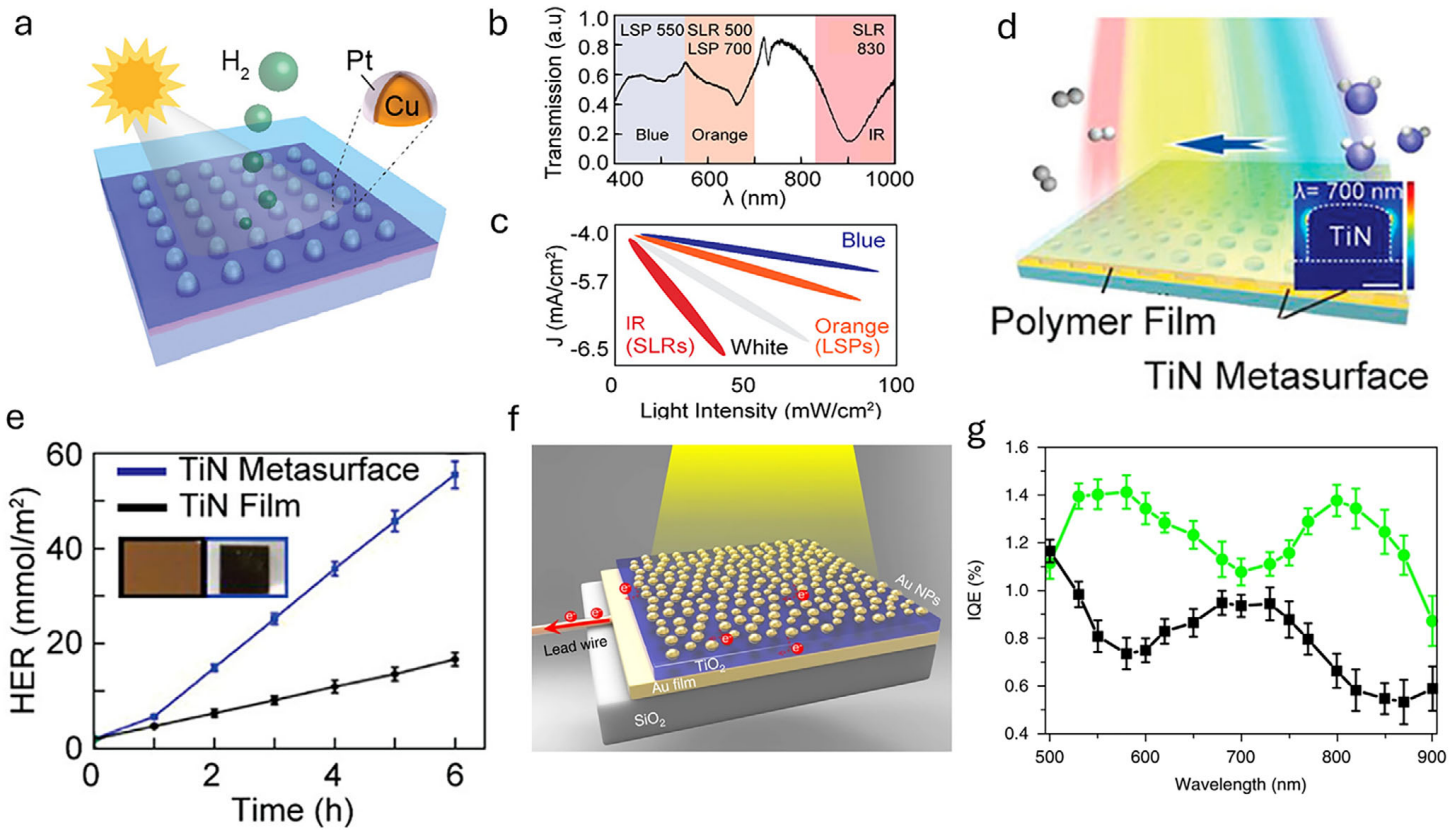
a) Schematic representation of Cu–Pt bimetallic core–shell nanoparticle arrays for photoelectrocatalytic hydrogen evolution reactions (HER). Reproduced with permission.^[^ ^38^ ^]^ Copyright 2021, ACS publication. b) The experimental transmission spectra of Cu@Pt nanoparticle (NP) lattices, highlighting the spectral ranges of different wavelengths (indicated by shaded areas). Reproduced with permission.^[^ ^38^ ^]^ Copyright 2021, ACS publication. c) The relationship between catalytic activity and light intensity across different wavelengths (blue, orange, white, IR). Reproduced with permission.^[^ ^38^ ^]^ Copyright 2021, ACS publication. d) Illustration of the titanium nitride (TiN) metasurface absorber integrated with a polymer film for enhanced HER. Reproduced with permission.^[^ ^77^ ^]^ Copyright 2021, ACS publication. e) HER performance comparison between TiN metasurface and TiN film over time. Reproduced with permission.^[^ ^77^ ^]^ Copyright 2021, ACS publication. f) Schematic of the Au‐NP/TiO_2_/Au‐film structure with partially embedded Au nanoparticles. Reproduced with permission.^[^ ^78^ ^]^ Copyright 2018, Springer Nature. g) Internal quantum efficiency (IQE) spectra of the Au‐NP/TiO_2_/Au‐film (green) and Au‐NP/TiO_2_ (black) photoelectrodes with a TiO_2_ thickness of 28 nm and Au‐NP inlaid depth of 7 nm. Reproduced with permission.^[^ ^78^ ^]^ Copyright 2018, Springer Nature.

An innovative approach involves the synthesis of bimetallic copper‐platinum (Cu‐Pt) core–shell nanoparticles arranged in periodic lattices (**Figure** **5**a). The combination of platinum's superior catalytic properties with copper's plasmonic characteristics creates a synergistic system that enhances HER activity under light illumination.^[^ ^38^ ^]^ The Cu‐Pt nanoparticles are organized into lattices that support surface lattice resonances (SLRs), which are collective oscillations of conduction electrons induced by the periodic arrangement. These SLRs generate intensified electromagnetic fields at the nanoparticle surfaces, leading to improved light absorption and more efficient electron generation and transfer‐key factors in boosting HER.^[^ ^38^ ^]^ By adjusting nanoparticle size and interparticle spacing, the plasmonic properties of the lattice can be tuned to optimize light absorption across a broader spectrum, including the near‐infrared (NIR) region (Figure 5b). This tunability surpasses the absorption limitations of traditional semiconductor photocatalysts like TiO₂, allowing for the harnessing of a larger portion of the solar spectrum.^[^ ^38^ ^]^ Experimental results demonstrate that the SLRs in these nanoparticle lattices significantly enhance HER activity compared to localized surface plasmons (LSPs) alone (Figure 5c). Under white‐light illumination, the HER efficiency increased by up to 60%, with catalytic activity more than doubling compared to systems utilizing only. The stronger localized electromagnetic fields associated with SLRs facilitate a higher rate of electron–hole pair generation and more efficient charge separation.

Overall, the periodic lattice structure enables highly tunable surface lattice resonances that enhance broadband light absorption, generate strong local electromagnetic fields to accelerate carrier generation, and produce localized heating. These effects work synergistically to improve photocatalytic hydrogen evolution performance compared to conventional catalysts.

Titanium nitride (TiN) metasurfaces have emerged as effective plasmon‐enhanced absorbers for HER in solar‐driven water splitting. TiN offers plasmonic properties similar to noble metals but with added benefits of thermal stability and cost‐effectiveness.^[^ ^77^ ^]^ The designed TiN metasurfaces as Figure 5d shows, achieve over 92% absorption in the visible light range of 400–750 nm. This broadband absorption is attributed to the excitation of LSPRs, which generate strong electromagnetic fields at the nanoscale. The LSPRs facilitate the generation of hot electrons, which are efficiently transferred to an integrated polymer photocatalyst. This plasmon‐enhanced charge separation results in a 300% increase in the HER compared to conventional TiN films (Figure 5e). TiN's high thermal stability ensures long‐term performance in solar‐energy systems. The combination of broadband absorption and robustness addresses common challenges in solar hydrogen production, making TiN metasurfaces suitable for sustainable photocatalytic applications. By integrating a polymer photocatalyst with the TiN metasurface, the system harnesses both plasmonic and thermal effects to enhance water splitting. This integration offers a novel approach to developing high‐efficiency solar‐energy harvesting systems, potentially leading to more effective hydrogen production methods. The advancements in metamaterial designs is ranging from bimetallic nanoparticle lattices and strong coupling structures to TiN metasurfaces, which highlight the crucial role of metamaterials in enhancing photocatalytic water splitting. By tailoring light–matter interactions through plasmonic effects, strong coupling, and optimized light absorption, these materials significantly improve catalytic efficiency. These innovations represent promising directions for sustainable hydrogen production, leveraging a larger portion of the solar spectrum and facilitating more efficient electron transfer mechanisms.

In this system, the nanostructured TiN metasurface combines plasmonic near‐field enhancement, efficient hot carrier injection, and photothermal heating. Together, these mechanisms improve light harvesting, charge separation, and reaction kinetics, resulting in significantly higher hydrogen evolution activity.

Another innovative strategy involves the use of gold nanoparticle (Au‐NP)/titanium dioxide (TiO_2_)/gold film structures that operate under strong coupling conditions (Figure 5f). This configuration leverages the interaction between LSPRs of Au nanoparticles and Fabry–Pérot nanocavity modes within the TiO_2_/Au film, resulting in hybrid modes that significantly enhance near‐field effects.^[^ ^78^ ^]^ The strong coupling between the LSPRs and cavity modes leads to the splitting of the absorption band into two distinct hybrid modes. These hybrid modes amplify electromagnetic fields near the nanoparticle surfaces, promoting more efficient generation and transfer of electrons necessary for water splitting (Figure 5g). The partially inlaid Au nanoparticles within the TiO_2_ layer atop a gold film facilitate efficient light harvesting by enhancing the interaction between plasmonic and cavity modes. This design achieves broadband absorption over a wide wavelength range. The incident photon‐to‐current conversion efficiency (IPCE) improves 11‐fold compared to configurations without the Au film, highlighting its superior performance in photocatalysis. The strong coupling promotes efficient hot electron transfer from the Au nanoparticles to TiO_2_, enhancing water oxidation efficiency. The overlapping hybrid modes facilitate improved charge separation and photocurrent generation, directly impacting the overall water‐splitting activity.

The hybrid cavity structure leverages strong plasmon–cavity coupling to intensify near‐field effects, facilitate hot carrier injection, and expand light absorption. These combined enhancements result in substantial improvements in photocurrent generation and overall water splitting efficiency.

## 5 Metamaterials for Organic Degradation

This section discusses three significant types of metamaterials used in photocatalysis for organic degradation: nanoporous Cu‐based metamaterials, biomimetic TiO_2_‐based metamaterials, and chiral plasmonic metamaterials.

**Figure 6:**
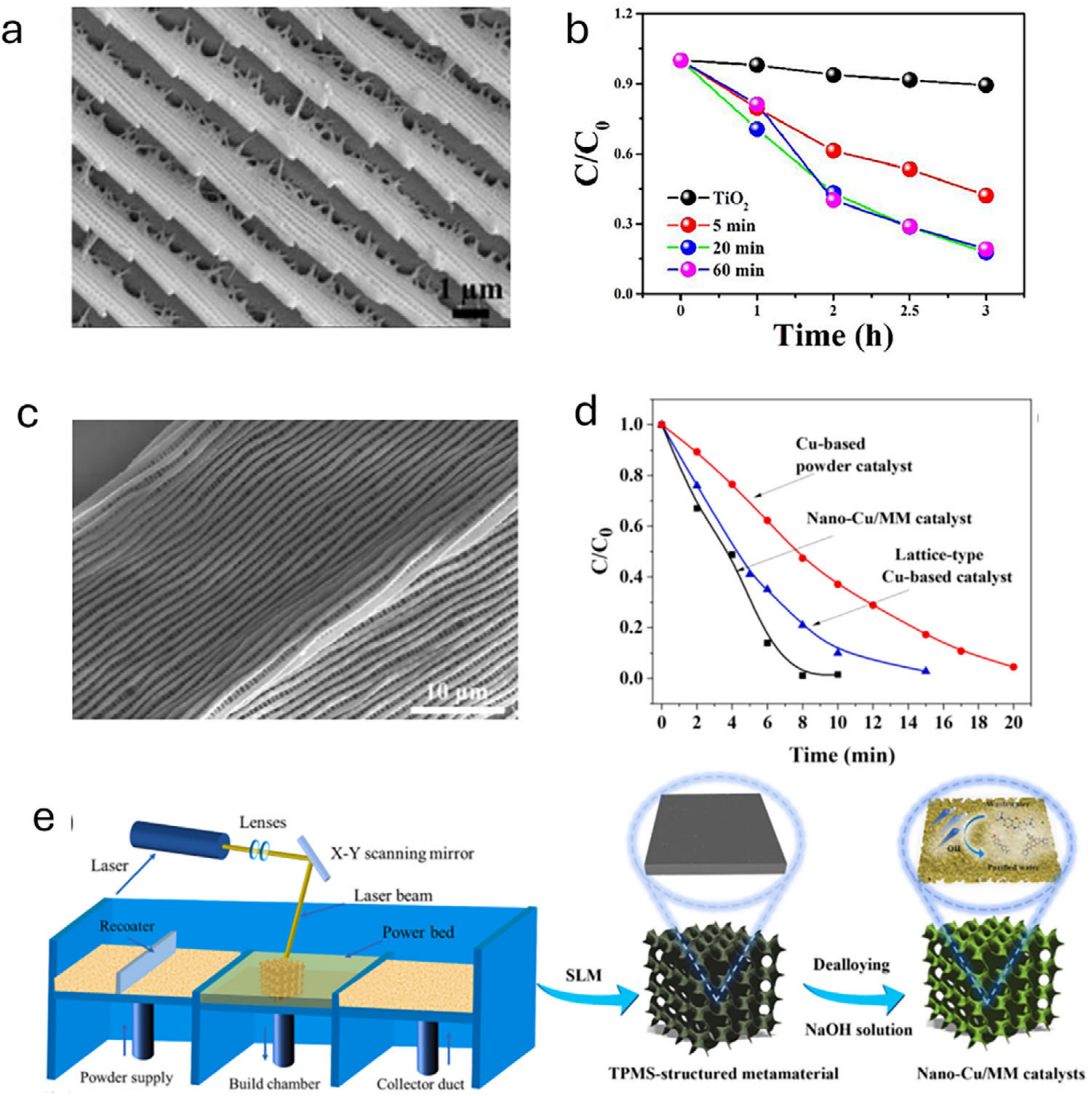
SEM image of a) butterfly wings and c) TiO_2_ replicas. Reproduced with permission.^[^ ^79^ ^]^ Copyright 2022, Elsevier. b) Performance of MB degradation by TiO₂ replicas with varying sputtering times. Reproduced with permission.^[^ ^79^ ^]^ Copyright 2022, Elsevier. d) Performance of degradation of MB with nano Cu/metamaterials and other control sample. Reproduced with permission.^[^ ^80^ ^]^ Copyright 2023, Elsevier. e) the process of fabrication of nano Cu/metamaterials. Reproduced with permission.^[^ ^80^ ^]^ Copyright 2023, Elsevier.

Biomimetic approaches have led to the development of TiO₂‐based metamaterials that mimic the hierarchical microstructures found in nature, such as butterfly wings.^[^ ^79^ ^]^ By replicating the intricate structures of butterfly wings, **Figure** **6**a shows the SEM image of butterfly wings, while Figure 6c presents the SEM image of TiO_2_ replicas, highlighting their structural similarity. These metamaterials exhibit enhanced light absorption due to their hierarchical architecture. This design increases the surface area and improves light–matter interactions, particularly in the visible spectrum, which is crucial for photocatalytic reactions.

TiO₂ is renowned for its high photocatalytic efficiency, chemical stability, and nontoxicity. The anatase phase of TiO₂, present in these biomimetic metamaterials, is particularly effective in photocatalysis. Furthermore, Ti^3^⁺ doping broadens the absorption spectrum into the visible light range, enhancing the photocatalyst's effectiveness under solar irradiation. Studies have shown that these TiO₂ replicas can achieve up to 90% degradation of MB after 3 h of sunlight exposure (Figure 6b), significantly outperforming conventional TiO₂ thin films.

In summary, the key design advantage lies in the bio‐inspired hierarchical microarchitecture, which provides both a large surface area and efficient light confinement via photonic light trapping. Together with Ti^3^⁺‐induced visible‐light absorption, these features greatly enhance light–matter interaction, increase charge carrier generation, and facilitate faster surface reactions. Consequently, these metamaterials achieve substantially higher reaction rates, improved quantum efficiencies, and accelerated pollutant conversion under solar irradiation compared to unstructured TiO₂, delivering superior photocatalytic performance with lower energy input.

Nanoporous Cu‐based metamaterials have gained attention as effective catalysts in advanced oxidation processes (AOPs), particularly for degrading complex organic pollutants in wastewater. Figure 6e shows the procedure to synthesis nano‐Cu/metamaterials catalysts. The nanoporous structure of Cu‐based metamaterials provides a high surface area and abundant active sites, facilitating effective mass transfer. These structures are often designed using triply periodic minimal surfaces (TPMS), achieved through selective laser melting and chemical dealloying techniques. The TPMS design not only enhances mechanical properties but also creates extensive pathways for reactants to access active sites.^[^ ^80^ ^]^ In Fenton‐like AOPs, nanoporous Cu‐based metamaterials catalyze the decomposition of hydrogen peroxide (H_2_O_2_) to generate hydroxyl radicals (•OH), potent oxidants capable of breaking down stable organic pollutants. The adjustable valence states of copper facilitate redox reactions, enhancing the production of •OH radicals. Experimental studies have demonstrated remarkable results, such as achieving up to 99% degradation of MB within just 10 min (Figure 6d). These catalysts have proven effective in degrading a variety of pollutants, including dyes like methyl orange and Rhb, and pharmaceuticals such as tetracycline.

In summary, the key feature of the nanoporous Cu‐based metamaterials is their precisely engineered 3D TPMS structure, which provides an interconnected mass transport channels. This architecture exposes a large number of Cu active sites and promotes efficient diffusion of both hydrogen peroxide and organic pollutants throughout the catalytic network. The performance enhancement is primarily driven by the Fenton‐like mechanism, where Cu⁰ and Cu(I) sites catalyze the decomposition of H₂O₂ to generate highly reactive hydroxyl radicals that rapidly degrade organic contaminants. In addition, the hierarchical porosity ensures uniform distribution of reactants and minimizes transport limitations, thereby accelerating the degradation process. As a result, these metamaterial catalysts achieve extremely high degradation efficiencies within short reaction times while maintaining excellent recyclability and durability, making them highly suitable for scalable industrial water treatment applications.

**Figure 7:**
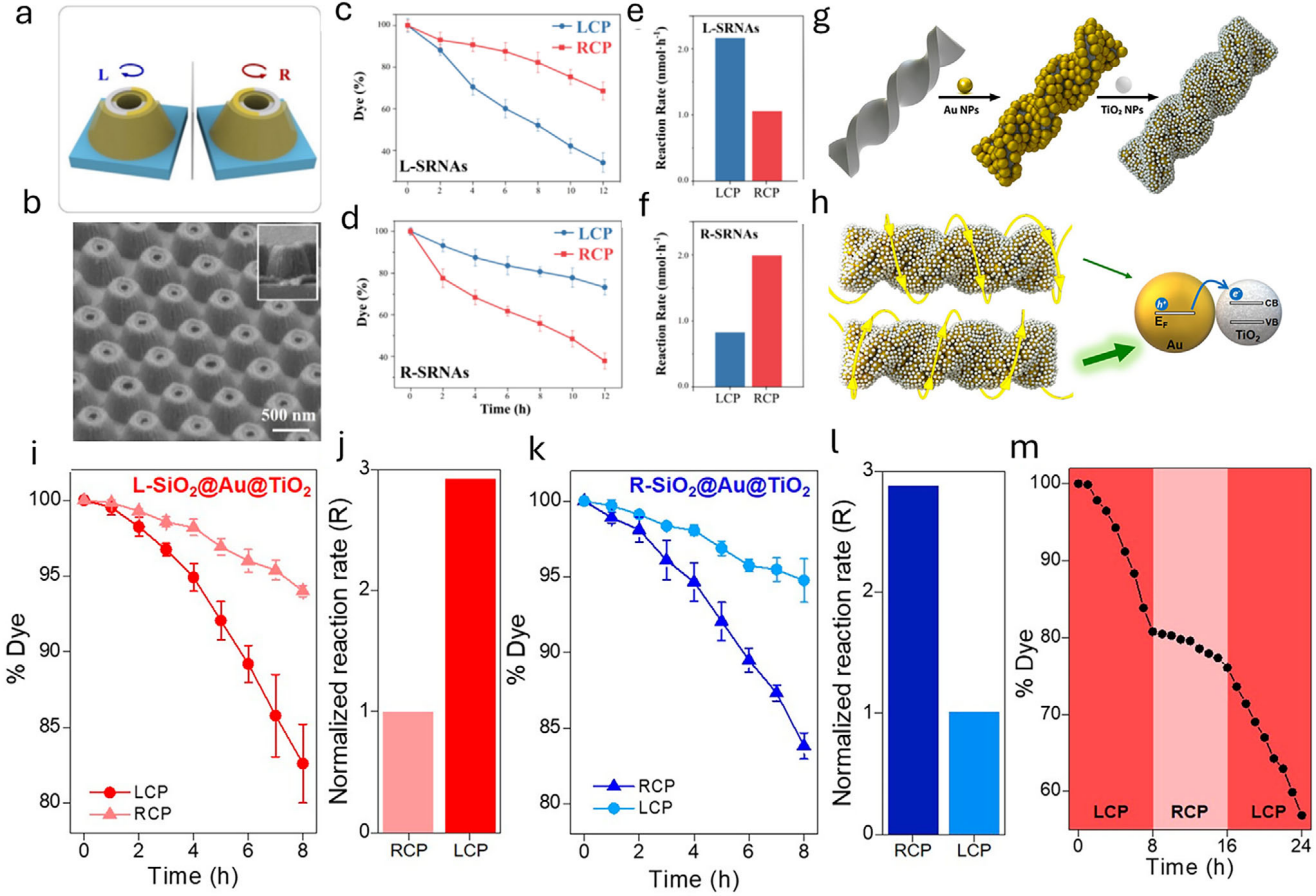
a) A schematic representation showing the mirrored chiral enantiomers of SRNAs. Reproduced with permission.^[^ ^58^ ^]^ Copyright 2025, Elsevier. b) SEM image showing the continuous structure of R‐SRNAs. Reproduced with permission.^[^ ^58^ ^]^ Copyright 2025, Elsevier. c) Photocatalytic degradation profiles of RhB using L‐SRNAs under LCP and RCP light illumination, and e) the corresponding normalized reaction rates. Reproduced with permission.^[^ ^58^ ^]^ Copyright 2025, Elsevier. d) Degradation profiles of RhB using R‐SRNAs catalysts under RCP and LCP light illumination, and f) the corresponding normalized reaction rates. Reproduced with permission.^[^ ^58^ ^]^ Copyright 2025, Elsevier. g) The adsorption process of gold (Au) and titanium dioxide (TiO₂) nanoparticles onto chiral silicon dioxide (SiO₂) nanoribbons. Reproduced with permission.^[^ ^59^ ^]^ Copyright 2022, ACS publication. h) The asymmetric interaction between a left‐handed (L) hybrid structure and circularly polarized light, highlighting the differing responses to left‐ and right‐circularly polarized light (LCP and RCP). i,k) Graphs depicting the photodegradation of RhB in the presence of a chiral catalyst under exposure to left‐ or right‐circularly polarized light (LCP or RCP). Reproduced with permission.^[^ ^59^ ^]^ Copyright 2022, ACS publication. j, l) Normalized reaction rates calculated from the photodegradation data. Reproduced with permission.^[^ ^59^ ^]^ Copyright 2022, ACS publication. m) Photodegradation performance of RhB when using left‐handed SiO₂@Au@TiO₂ nanoribbons with various polarizers. Reproduced with permission.^[^ ^59^ ^]^ Copyright 2022, ACS publication.

Chiral plasmonic metamaterials represent a cutting‐edge development in photocatalysis, offering polarization‐sensitive degradation of organic pollutants through the interaction of chiral structures with CPL.^[^ ^25^, ^57^, ^58^, ^59^, ^60^, ^61^, ^62^ ^]^ Studies have introduced chiral metamaterials such as Swiss roll nanoarrays (SRNAs) and chiral Au‐TiO₂ nanohybrids that exhibit strong chiroptical responses in the visible region.^[^ ^59^ ^]^ These materials demonstrate enhanced photocatalytic degradation of organic dyes when the chirality of the metamaterial matches the handedness of the CPL:

**Figure** **7**a illustrates the mirrored chiral SRNAs enantiomers. The left‐handed SRNAs (L‐SRNAs) exhibit significantly higher degradation rates of RhB under left‐handed CPL compared to right‐handed CPL (Figure 7c,e), while the opposite is observed for the right‐handed SRNAs (R‐SRNAs) (Figure 7d,f), as shown in the SEM image in Figure 7b. This chirality matching leads to a 2.05–2.42‐fold increase in the degradation rate (Figure 7e,f).^[^ ^58^, ^59^ ^]^

Moreover, as Figure 7g shows the electrostatic adsorption of TiO_2_ and Au nanoparticles enables the formation of a chiral metal‐semiconductor photocatalyst, where hot electron injection across the Schottky barrier becomes sensitive to light polarization due to the chiroptical properties of the plasmon signal (Figure 7h).^[^ ^59^ ^]^ The chiral Au‐TiO₂ nanohybrids exhibit a remarkable 2.93‐fold increase in the RhB degradation rate when the helicity of the CPL aligns with the handedness of the chiral nanostructures (Figure 7j,l). The enhanced activity is attributed to the localized surface plasmon resonance effect in chiral nanostructures, which facilitates the asymmetric generation of hot carriers (energetic electrons and holes) under CPL. When the chirality of the light and the material match, there is increased excitation of plasmonic modes, leading to a higher density of hot carriers that participate in photocatalytic processes (Figure 7k,l). Figure 7m also confirms that the L‐SiO₂@Au@TiO₂ catalyst exhibits higher photocatalytic activity when matched with LCP light. These hot carriers enhance the generation of reactive oxygen species like hydroxyl radicals (•OH) and superoxide anions (•O₂⁻), which effectively degrade organic pollutants.

Although CISS has demonstrated around twofold enhancements in chiral metamaterials,^[^ ^58^ ^]^ its underlying mechanisms remain under investigation and have so far been validated only in a few model systems with strong structure–reaction dependencies. Likewise, dye‐degradation assays—while convenient for initial activity screening—involve relatively simple oxidative pathways and potential dye photolysis, making them poor proxies for industrially relevant processes such as CO₂ reduction or H₂ evolution. To translate these chiral photocatalysis strategies into real‐world applications, catalyst performance must be validated in more demanding, multi‐electron or proton transfer reactions, assessing both efficiency and selectivity under practical conditions.

## 6 Metamaterials for Hydrogen Evolution and Water Evaporation

**Figure 8:**
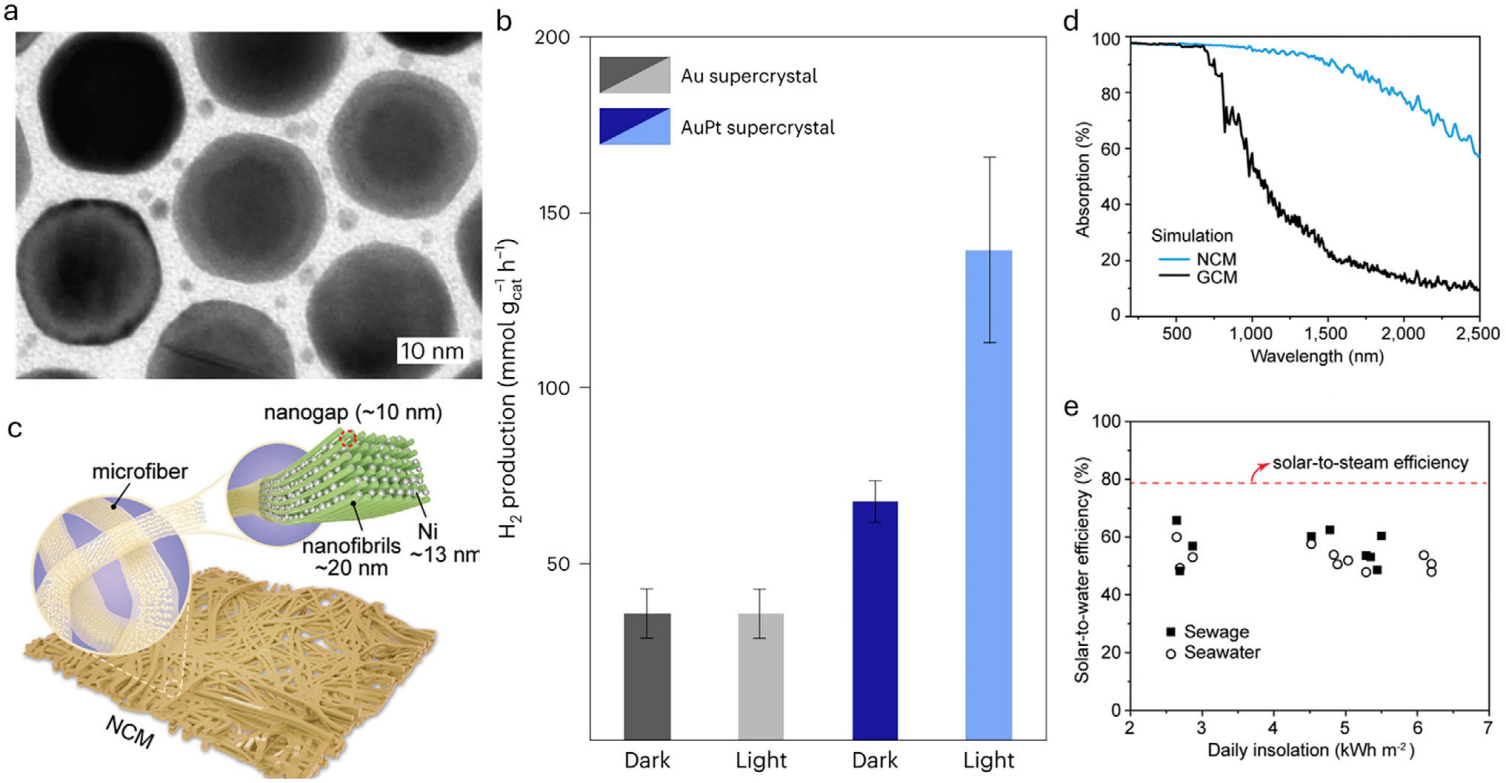
a) TEM images of a bimetallic 2D AuPt supercrystal, PtNPs (3 nm) are hosted at the interparticle gap (3.5 nm) between the 22 nm AuNPs. Reproduced with permission.^[^ ^18^ ^]^ Copyright 2023, Springer Nature. b) H_2_ generation rate normalized by the total mass of catalyst in both conditions, dark and light, for Au and AuPt supercrystals. Reproduced with permission.^[^ ^18^ ^]^ Copyright 2023, Springer Nature. c) A schematic illustration of the hierarchical structures of NCM, emphasizing the space‐confinement effect on nickel (Ni) nanoparticles located between crystalline nanofibrils. Reproduced with permission.^[^ ^81^ ^]^ Copyright 2020, Wiley‐VCH. d) The simulated optical absorption spectra for NCM and GCM materials, averaged over both transverse electric (TE) and transverse magnetic (TM) polarizations, calculated using the finite element method (FEM). Reproduced with permission.^[^ ^81^ ^]^ Copyright 2020, Wiley‐VCH. e) The solar‐to‐water evaporation efficiencies achieved during the purification of sewage and seawater. Reproduced with permission.^[^ ^81^ ^]^ Copyright 2020, Wiley‐VCH.

A two‐dimensional superlattice composed of gold (Au) nanoparticles, with platinum (Pt) nanoparticles placed in the gaps between the particles were studied by Matias et al.^[^ ^18^ ^]^ **Figure** **8**a presents a transmission electron microscopy (TEM) image of the material, showcasing platinum (Pt) nanoparticles with diameters of 3 nm and gold (Au) nanoparticles with diameters of 22 nm. The gap between the two kinds of nanoparticles is ≈3.5 nm. This structure combines the plasmonic‐enhanced optical properties of Au with the catalytic activity of Pt for hydrogen evolution. The superlattice is optimized to generate strong localized electric fields (hotspots), which concentrate light energy at the catalytic sites, significantly enhancing the photocatalytic performance. Under visible light, these superlattices exhibit a hydrogen production rate of up to 139 mmol g⁻¹ h⁻¹ through formic acid dehydrogenation (Figure 8b). This high performance is attributed to the effective plasmonic enhancement provided by the well‐structured bimetallic superlattice, ensuring that Pt nanoparticles receive concentrated electromagnetic energy, significantly boosting catalytic efficiency. The Au–Pt superlattice metamaterial integrates precise spatial organization of plasmonic and catalytic components to maximize light harvesting and catalytic efficiency. The critical design feature is the sub‐4 nm interparticle spacing between Au and Pt nanoparticles, which enables strong plasmonic coupling and highly localized electromagnetic fields concentrated at the Pt catalytic sites. This near‐field enhancement amplifies light absorption and significantly increases the generation of hot carriers, which are directly involved in the catalytic dehydrogenation of formic acid. Furthermore, the structural tunability of the superlattice, including nanoparticle size, gap distance, and stacking layers, allows optimization of the plasmon resonance conditions to match the incident solar spectrum. As a result, the plasmon‐assisted hot electron transfer and concentrated local heating synergistically promote efficient hydrogen evolution, achieving high reaction rates under visible light.

In the field of water evaporation, the design of nickel‐cellulose hybrid metamaterials (NCM) offers a highly efficient approach for solar‐driven water purification (Figure 8c).^[^ ^81^ ^]^ The key structural feature lies in the nanoscale confinement of nickel nanoparticles (smaller than 40 nm) uniformly embedded within the porous cellulose nanofiber network through nano‐confinement synthesis. First, the interband transitions (IBTs) of nickel directly contribute to broadband light absorption enhancement, extending solar harvesting from visible to near‐infrared regions, and yielding a solar‐weighted absorptivity of up to 97.1% (Figure 5d), which corresponds to the optical absorption engineering mechanism. Second, the spatial confinement of metallic nanoparticles within the low‐refractive‐index cellulose matrix suppresses reflectance and scattering losses, ensuring more efficient photon trapping and light utilization. Third, the absorbed solar energy is effectively converted into localized photothermal heating at the water–vapor interface, which minimizes bulk thermal dissipation and improves heat localization consistent with the photothermal conversion mechanisms described in Section 2. In parallel, the porous cellulose framework provides continuous capillary water transport toward the heated interface, maintaining steady‐state evaporation under prolonged illumination. As a result, NCM achieves solar‐to‐water conversion efficiencies ranging from 47.9% to 65.8% under simulated solar illumination (Figure 8e). The system also demonstrates excellent long‐term operational stability and scalability, which highlights its practical potential for large‐scale solar desalination and wastewater treatment.

## 7 Future Outlook

**Table** **1** provides a concise summary of the various metamaterial architectures discussed above and their corresponding photocatalytic applications. To translate metamaterial‐enhanced photocatalysis from lab‐scale innovation to real‐world application, key challenges must be addressed. These include scalable fabrication, system integration, long‐term stability, and complex design optimization. The following sections outline promising directions to overcome these hurdles and advance the field toward practical deployment.

**Table 1:** Overview of metamaterials for various applications in photocatalysis.

Material	Principle	Application	Performance	Ref.
Ni‐based photothermal metamaterial	Enhances local electric field via nickel plasmonic resonance, promoting CO₂ adsorption and activation	CO₂ reduction	CO₂ conversion rate of 516.9 mmol g⁻¹ h⁻¹ with 90% CO selectivity, stable for 10 h	[71]
Stacked plasmonic metamaterial ASA‐c‐Ag8Cu1	Ultra‐broadband light absorption, surface temperature > 300 °C, periodic Au triangular array creates strong local electric fields, promoting plasmonic “hot‐electron” injection	CO₂ reduction	CO 1106 mmol m^−2^ and CH_4_ 301 mmol m^−2^ (24 h)	[76]
Bi₂S₃/P‐ZnO S‐scheme metamaterial	S‐scheme heterojunction for efficient charge separation and enhanced light absorption	CO₂ reduction	3.45× higher CO production, 4.65× higher CH₄ production than P‐ZnO alone	[42]
Biomimetic TiO₂ structure	Increases light absorption for enhanced photocatalytic activity	Organic pollutant degradation	Achieves 90% MB degradation under sunlight within 3 h	[79]
Nanoporous Cu structure	Generates •OH radicals in Fenton‐like oxidation to enhance organic pollutant degradation	Organic pollutant degradation	Degrades 99% of MB in 10 min	[80]
Swiss roll metamaterial	Matches chirality with circularly polarized light to increase degradation rate of organic pollutants	Organic pollutant degradation	Enhances RhB degradation rate by 2.05–2.42× under chirality‐matched light	[58]
Chiral SiO₂@Au@TiO₂	Plasmonic enhancement and Polarization‐dependent “hot” electron generation and injection into TiO₂ under circularly polarized light (CPL)	Organic pollutant degradation	L‐SiO₂@Au@TiO₂ under L‐CPL shows a 2.93× higher photodegradation rate vs R‐CPL	[59]
Cu‐Pt core–shell structure	Uses surface lattice resonance to intensify electromagnetic field, boosting HER efficiency	Water splitting	Increases HER efficiency by over 60% under white light	[38]
TiN metasurface	Achieves broadband absorption in the visible spectrum, improving thermal stability and long‐term performance	Water splitting	HER performance 300% higher than conventional TiN film	[77]
Au‐NP/TiO₂/Au‐film structure	Promotes charge separation through strong coupling effects for enhanced photocatalytic efficiency	Water splitting	11× higher water splitting efficiency than configuration without Au film	[78]
Au‐Pt 2D superlattice	Uses plasmonic enhancement to boost light absorption for hydrogen production	H₂ generation	Hydrogen production rate of up to 139 mmol g⁻¹ h⁻¹ via formic acid dehydrogenation	[18]
Ni‐cellulose composite material	Utilizes nickel's broadband absorption for high solar‐thermal evaporation efficiency	Water evaporation	Solar‐to‐water conversion efficiency between 47.9% and 65.8%, suitable for wastewater and seawater purification	[81]

### 7.1 Scalable Manufacturing and Integration into Real‐World Systems

Metamaterials for solar‐driven photocatalysis have demonstrated remarkable performance enhancements at the laboratory scale, but translating these advances into real‐world systems remains challenging. A major hurdle is the lack of economical, high‐throughput fabrication methods for large‐area metamaterial structures.^[^ ^3^, ^8^, ^24^, ^34^ ^]^ Conventional nanofabrication (e.g., electron‐beam lithography) is too slow and costly for scalable production. Recent research has therefore explored alternative manufacturing techniques.^[^ ^3^, ^8^, ^24^, ^34^, ^82^, ^83^ ^]^ For instance, nanoimprint lithography has been used to pattern semiconductor metasurface photoelectrodes over wafer‐scale areas,^[^ ^24^ ^]^ bridging the gap between single‐nanostructure studies and practical device sizes. Self‐assembly and colloidal approaches are also emerging, whereby nanoparticles or “meta‐atoms” organize into periodic architectures (“meta‐colloid” clusters) that exhibit metamaterial properties.^[^ ^24^ ^]^ Such bottom‐up methods, combined with roll‐to‐roll processing or template‐assisted deposition, could dramatically improve throughput and reduce costs.

In addition to planar metasurfaces, 3D printing is opening new avenues for integrating metamaterial concepts into catalytic reactors. A striking example is a wood‐inspired lattice metamaterial catalyst 3D‐printed in stainless steel and coated with active cobalt.^[^ ^84^ ^]^ This structure achieved high mechanical strength, enhanced mass transport, and improved reaction kinetics for water purification, illustrating how architected materials can be scaled to centimeter dimensions without sacrificing functionality. Although it itself does not constitute a photonic metamaterial, it underscores the potential of additive manufacturing to produce catalyst supports with tailored geometry. Looking ahead, similar 3D fabrication strategies might be adapted to create hierarchical photonic structures (e.g., photonic crystal fibers or monoliths) that maximize light delivery to photocatalytic sites in large reactors.

Effective integration of metamaterial photocatalysts into real‐world systems will require close collaboration between materials scientists and process engineers. Unlike conventional photocatalysts (often used as powders or thin films), metamaterial structures may need specialized reactor designs. For example, a metamaterial‐coated photoelectrode for solar water splitting must be incorporated into an electrochemical cell without obscuring its active surface.^[^ ^84^ ^]^ Likewise, metamaterial‐enhanced photocatalytic surfaces could line the walls of flow reactors or be combined with light‐concentrating optics. In this regard, concepts from solar concentrator technology (such as compound parabolic collectors) could be coupled with metamaterial absorbers to ensure efficient utilization of sunlight in large volumes. Modular reactor designs might employ removable metamaterial panels or inserts that can be swapped in as catalytic cartridges.

To realize these integrations, researchers are developing design approaches that maintain performance uniformity over large areas and non‐flat geometries. Techniques like substrate conformal lithography and flexible metasurface patterning have been studied to transfer nanopatterns onto curved or irregular surfaces.^[^ ^84^ ^]^ Moreover, advanced simulation tools are helping to scale up designs. Recent efforts highlight the use of neural‐network‐based modelling and inverse design to optimize metasurface performance on expanded footprints.^[^ ^85^ ^]^ By reducing computational cost, these approaches enable the design of meter‐scale metamaterial components with minimal loss of efficiency at the edges or between tiled units. Such innovations, alongside progress in manufacturing, point toward a future where metamaterial photocatalysts can be produced in rolls or sheets and integrated much like conventional catalytic liners or solar absorber panels.

In summary, achieving scalable manufacturing and integration will demand interdisciplinary efforts. Nanofabrication experts are needed to refine large‐area patterning and printing techniques, while chemical engineers must adapt reactor configurations to accommodate and leverage metamaterial components. Material cost and manufacturability considerations should be factored into metamaterial design from the outset, favoring simpler architectures or inexpensive base materials (e.g., aluminum or plastics with metamaterial coatings).^[^ ^86^ ^]^ By addressing these practicalities, the field can move beyond proof‐of‐concept devices toward deployable photocatalytic systems that exploit the full power of metamaterials under real sunlight and operating conditions.

### 7.2 Environmental and Long‐Term Stability in Harsh Conditions

Stability under operational conditions is a critical concern for any photocatalyst, and the complex nanostructures of metamaterials introduce additional challenges. Solar‐driven reactions frequently occur under harsh conditions. These include strong ultraviolet exposure, reactive radical species, contact with water or electrolytes, and repeated thermal cycling. Such conditions can gradually degrade metamaterial performance. Indeed, the poor long‐term durability of many photocatalytic materials has been a key factor limiting their practical application.^[^ ^87^ ^]^ For metamaterials, which commonly incorporate nanoscale metals or semiconductors, maintaining structural and chemical integrity is paramount. For example, plasmonic metamaterial catalysts using silver or aluminum can suffer oxidation, corrosion, or agglomeration of nanostructures upon prolonged UV exposure and contact with water or oxygen. Even gold, while chemically inert, may undergo surface restructuring or coarsening at elevated temperatures, blunting the nanoscale features responsible for its optical response.

To improve robustness, researchers are exploring material substitutions and protective strategies. One promising direction is the use of refractory plasmonic materials such as titanium nitride (TiN), zirconium nitride, or tungsten, in place of traditional noble metals.^[^ ^54^ ^]^ These materials support plasmonic resonances and strong light absorption, yet exhibit much higher melting points, hardness, and resistance to oxidation. A recent study demonstrated an ultrabroadband absorber based on a TiN/TiO_2_ heterostructure that retained performance at temperatures and conditions that would damage conventional plasmonic.^[^ ^54^ ^]^ Similarly, high‐index dielectrics (e.g., silicon or gallium nitride) can be structured to produce Mie resonances or photonic band effects without relying on metal at all, thereby avoiding issues of metal corrosion. An example is gallium phosphide (GaP) metasurface photoelectrodes, which were chosen not only for suitable bandgap but also for their stability in aqueous electrolytes.^[^ ^24^ ^]^

Another approach is encapsulation or surface engineering of metamaterials to shield them from the environment. Thin oxide or carbon coatings can sometimes protect metallic nanostructures from oxidation while allowing light to pass. Core–shell nanoparticle architectures (for instance, a plasmonic core with a thin silica shell) have shown improved stability in photocatalytic conditions, as the shell can prevent direct contact with corrosive species. In the context of metamaterials, implementing such coatings uniformly over a periodic nanostructure is nontrivial but could significantly extend lifetime. Likewise, careful choice of support substrate can help using chemically inert and thermally conductive supports (like sapphire or ceramics) may mitigate thermal hotspots and prevent catalysis‐induced stress cracking.

Long‐term stability testing is becoming an essential component of metamaterial photocatalysis research. Recent reports have started to include multi‐cycle reaction tests and extended illumination trials. For example, a TiN/TiO_2_ metamaterial photocatalyst was shown to sustain enhanced H₂ evolution over repeated on/off light cycles, outperforming an unstructured catalyst in both activity and endurance.^[^ ^54^ ^]^ Such studies point to another benefit of metamaterials: by concentrating light and reactions at specific “hot spots,” they might enable lower overall light intensity or shorter reaction times for the same output, potentially reducing cumulative stress on the material. Nonetheless, systematic aging experiments (e.g., hundreds of hours of continuous solar illumination, or outdoor exposure tests) are still rarely reported and will be crucial for understanding failure modes. Photocatalytic reactors often face fouling (deposit buildup) and abrasion (from fluid flow); how nanostructured metamaterial surfaces handle these issues remains to be seen.

Future research directions should prioritize stability engineering on equal footing with performance. One vision is the development of “rugged” metamaterials: architectures designed with damage mitigation in mind. This could include self‐cleaning hydrophobic coatings to prevent fouling, sacrificial layers that can be periodically peeled off and renewed, or modular designs where a degraded metamaterial component can be replaced without discarding the entire system. Another important direction involves testing metamaterials under realistic solar spectra and weather conditions. This includes assessing photochemical changes caused by UV exposure or thermal stress from day‐to‐night temperature fluctuations. Insights from corrosion science and mechanical durability studies, such as fatigue testing, will be critical. Addressing these challenges requires interdisciplinary collaboration. By working with chemical engineers and environmental scientists, materials researchers can identify which failure mechanisms (photocorrosion, oxidation, mechanical wear, etc.) are most problematic in practical operation, and then tailor the chemistry and structure of metamaterial catalysts to counteract those specific issues.

Ultimately, improving the environmental and long‐term stability of metamaterial photocatalysts is essential for their commercialization. Success in this area will enable future metamaterial‐enhanced reactors to operate reliably for months or even years under sunlight, maintaining high performance with minimal maintenance. Achieving such stability may involve trade‐offs, such as modest reductions in peak efficiency to improve durability. However, these compromises can be optimized through strategic choices in materials selection, protective coatings, and structural reinforcement. The continued evolution of stable, sunlight‐hardy metamaterials will pave the way for real‐world solar‐to‐chemical energy conversion systems that are not only efficient but also robust and trustworthy in the field.

### 7.3 Advanced Techniques in Metamaterials

As metamaterial design spaces become ever more complex and high‐dimensional, advanced computational methods are increasingly indispensable.^[^ ^88^, ^89^ ^]^ Inverse‐design strategies now allow researchers to specify a desired optical or catalytic response and let algorithms determine the required nano‐geometry. For example, genetic algorithms (GA) and evolutionary optimizers have been applied to metasurfaces to discover counter‐intuitive unit‐cell arrangements that maximize broadband absorption or tailor spectral coloration.^[^ ^90^ ^]^ In one study, expanding the number of “meta‐atoms” per unit cell and using a GA‐based optimizer yielded metasurfaces with dramatically improved absorption and color‐gamut performance.^[^ ^90^ ^]^ Similarly, deep neural networks have been trained to map between geometric parameters and optical spectra, enabling true inverse design of plasmonic metasurfaces. Ramakrishna et al. demonstrated a bi‐directional deep network that could infer subwavelength nanoparticle geometries from far‐field spectra and vice versa, effectively solving the inverse problem and allowing rapid on‐demand design of complex metasurface elements.^[^ ^91^ ^]^

Machine learning models serve as fast surrogates for expensive electromagnetic simulations, massively accelerating the search for optimal designs.^[^ ^92^ ^]^ For instance, convolutional neural networks (CNNs) or graph neural networks can predict a metamaterial's band structure or absorption spectrum from its geometry in milliseconds, after which optimization routines (GAs, particle swarm, gradient‐based methods, etc.) refine the design. Tran et al. recently used a CNN in tandem with a conditional variational autoencoder (cVAE) to inverse‐design photonic metamaterial unit cells with arbitrary target bandgap characteristics.^[^ ^93^ ^]^ In their framework, the CNN first learns to predict the bandgap width and center frequency of a structure, and then the cVAE generates new unit‐cell topologies that achieve a user‐specified bandgap. This data‐driven approach bypasses lengthy trial‐and‐error scans and directly yields feasible designs for custom photonic bandgaps.^[^ ^93^ ^]^ More generally, nature‐inspired artificial intelligence (AI) methods have proven effective for metamaterial lattices: genetic algorithms combined with neural‐network surrogate models efficiently explore vast parameter spaces. As Cerniauskas et al. note, GA schemes are widely used in metasurface optimization, and when paired with ML (e.g., neural‐network surrogates) they can uncover novel lattice structures that satisfy complex multi‐constraint objectives.^[^ ^9^ ^]^

Beyond purely planar designs, emerging metamaterial concepts like optical chirality are also being integrated into AI‐driven workflows. Chiral plasmonic metamaterials, which lack mirror symmetry and distinguish left‐ and right‐circularly polarized light, open new degrees of freedom for photocatalysis (e.g., by inducing spin‐selective carrier dynamics). At the microscopic level, Ai et al. showed that introducing an atomic‐scale helical twist in ZnO crystals (via chiral templating) created an effective spin filter: the resulting chiral ZnO exhibited spin‐polarized photocarriers and roughly twofold higher O_2_ evolution under illumination, compared to achiral ZnO.^[^ ^25^ ^]^ At the metasurface level, engineered anisotropic or twisted nano‐antenna arrays can produce strong circular dichroism and circular‐polarization conversion. Designing such 3D chiral structures with multiple free parameters (e.g., twist angle, layer sequence, strain) is well‐suited to AI algorithms. Zhang et al. demonstrated an AI‐guided approach to chiral film design using a generative adversarial network: a trained forward neural network first predicts the circular dichroism (CD) spectrum of a candidate film from its layer parameters, and then a neural “generator” proposes new layer parameters that move the spectrum toward a desired CD target.^[^ ^62^ ^]^ By iterating this generator–discriminator loop, the system converges on film geometries that achieve user‐defined chiroptical indices. In this way, AI can efficiently navigate the high‐dimensional design space of chiral metamaterials and films, locating structures that might be impossible to find by intuition alone.^[^ ^62^ ^]^

AI techniques are likewise being applied to LSPR and photonic band structures in tandem. Dense plasmonic metasurfaces create intense near‐fields (hotspots) that can drive surface reactions, and AI optimization can tune their geometry and material composition for maximal field enhancement at catalytic sites. Simultaneously, many metamaterial architectures aim to introduce photonic bandgaps or slow‐light effects to trap light; these objectives can be incorporated into the inverse design as well. For example, as noted above, deep learning frameworks have been developed that directly output structures with prescribed bandgap frequencies.^[^ ^93^ ^]^ By combining multiple objectives (such as matching an LSPR to a catalyst's bandgap while also opening a photonic bandgap), these multiphysics algorithms can propose unconventional, multiscale architectures. Such hybrid designs (e.g., multilayer metasurfaces with embedded dielectric photonic crystals and metal nano‐antennas) would be nearly impossible to optimize by hand but become tractable with AI‐driven search. In short, advanced algorithms enable exploration of structural motifs—including nonperiodic or hierarchical unit cells—that defy simple pattern intuition, while ensuring that both optical and catalytic criteria are met.

Looking forward, these advanced methodologies promise to dramatically shorten the design–test cycle for metamaterials. One vision is to integrate AI with high‐throughput fabrication and characterization. In this scenario, an AI model proposes a set of candidate metastructures, a robotic nanofabrication platform prints or assembles them in parallel, and automated spectroscopy measures their response–feeding the results back into the learning algorithm for further improvement. Such closed‐loop experimental platforms are already emerging in related fields and could soon apply to photocatalytic metamaterials. In practice, this means the AI continuously refines its own surrogate models and generator networks using real‐world data, ultimately converging on high‐performance designs much faster than traditional Edisonian approaches. In summary, the convergence of AI‐driven inverse design with advanced metamaterial concepts (chirality, LSPR engineering, bandgap control, etc.) is giving rise to a new design paradigm. This paradigm emphasizes methodology: algorithms discover novel nano‐architectures that engineer light–matter interactions in precise ways. The expected outcome is a generation of photocatalytic metamaterials—potentially involving nonintuitive, multifunctional, or reconfigurable geometries—that maximize solar‐to‐chemical conversion in ways human designers would find very difficult to uncover. By embracing these advanced techniques, researchers aim to explore the full design landscape of metamaterials and accelerate the discovery of practical, optimized structures for solar fuels and environmental catalysis.

## 8 Conclusion

Metamaterials have opened new frontiers in the rational design of photocatalytic systems by enabling precise control over light harvesting, charge dynamics, and surface chemistry. Their unique optical responses have significantly enhanced the performance of CO₂ reduction, hydrogen evolution, water splitting, water evaporation and pollutant degradation reactions under solar irradiation. However, several practical challenges remain, particularly in relation to manufacturing scalability, material stability, and reactor‐level integration. Overcoming these barriers will require collaborative efforts across materials science, chemical engineering, and optical physics. The adoption of artificial intelligence for inverse design and performance prediction is expected to accelerate progress, enabling the discovery of unconventional architectures and material combinations. With these advances, metamaterial‐enhanced photocatalysts are expected to play a critical role in the development of next‐generation clean energy and environmental remediation technologies.

## Conflict of Interest

The authors declare no conflict of interest.

## Data Availability

Data availability is not applicable to this article as no new data were created or analyzed in this study.
